# The Nutritional Profiles of Five Important Edible Insect Species From West Africa—An Analytical and Literature Synthesis

**DOI:** 10.3389/fnut.2021.792941

**Published:** 2021-12-03

**Authors:** Jacob P. Anankware, Benjamin J. Roberts, Xavier Cheseto, Isaac Osuga, Vincent Savolainen, C. M. Collins

**Affiliations:** ^1^Department of Horticulture and Crop Production, University of Energy and Natural Resources, Sunyani, Ghana; ^2^Georgina Mace Centre for the Living Planet, Faculty of Natural Sciences, Imperial College London, London, United Kingdom; ^3^Department of Chemical and Behavioural Sciences, International Centre for Insect Physiology and Ecology, Nairobi, Kenya; ^4^Department of Animal Sciences, Jomo Kenyatta University of Agriculture and Technology, Nairobi, Kenya

**Keywords:** entomophagy, nutrition, food security, *Hermetia illucens*, *Musca domestica*, *Rhynchophorus phoenicis*, *Cirina butyrospermi*, *Macrotermes bellicosus*

## Abstract

**Background:** Undernutrition is a prevalent, serious, and growing concern, particularly in developing countries. Entomophagy—the human consumption of edible insects, is a historical and culturally established practice in many regions. Increasing consumption of nutritious insect meal is a possible combative strategy and can promote sustainable food security. However, the nutritional literature frequently lacks consensus, with interspecific differences in the nutrient content of edible insects generally being poorly resolved.

**Aims and methods:** Here we present full proximate and fatty acid profiles for five edible insect species of socio-economic importance in West Africa: *Hermetia illucens* (black soldier fly), *Musca domestica* (house fly), *Rhynchophorus phoenicis* (African palm weevil), *Cirina butyrospermi* (shea tree caterpillar), and Mac*rotermes bellicosus* (African termite). These original profiles, which can be used in future research, are combined with literature-derived proximate, fatty acid, and amino acid profiles to analyse interspecific differences in nutrient content.

**Results:** Interspecific differences in ash (minerals), crude protein, and crude fat contents were substantial. Highest ash content was found in *H. illucens* and *M. domestica* (~10 and 7.5% of dry matter, respectively), highest crude protein was found in *C. butyrospermi* and *M. domestica* (~60% of dry matter), whilst highest crude fat was found in *R. phoenicis* (~55% of dry matter). The fatty acid profile of *H. illucens* was differentiated from the other four species, forming its own cluster in a principal component analysis characterized by high saturated fatty acid content. *Cirina butyrospermi* had by far the highest poly-unsaturated fatty acid content at around 35% of its total fatty acids, with α-linolenic acid particularly represented. Amino acid analyses revealed that all five species sufficiently met human essential amino acid requirements, although *C. butyrospermi* was slightly limited in leucine and methionine content.

**Discussion:** The nutritional profiles of these five edible insect species compare favorably to beef and can meet human requirements, promoting entomophagy's utility in combatting undernutrition. In particular, *C. butyrospermi* may provide a source of essential poly-unsaturated fatty acids, bringing many health benefits. This, along with its high protein content, indicates that this species is worthy of more attention in the nutritional literature, which has thus-far been lacking.

## Introduction

Two prominent issues facing global development are that of widespread undernutrition and poverty (1). A recent report by the Food and Agriculture Organization (2) estimated the number of people globally experiencing severe food insecurity at 750 million, a number which rises to two billion when moderate food insecurity is considered, with over 20% of children under five showing stunting. These issues particularly affect low-income countries, where nutritious diets are widely unaffordable and staple foods, often of low nutritional value, are relied upon (2). Food insecurity can have devastating health impacts, with undernutrition thought to be the leading cause of child mortality (3). Furthermore, early-life undernutrition has been linked to later-life issues, including hypertension, diabetes, and cardiovascular disease (3–5). Food insecurity has even been implicated in reducing immune function, increasing non-communicable disease burden, and altering gut microbiome (6, 7). The economic burden of diet-related mortality and non-communicable disease is expected to exceed 1.3 trillion dollars annually by 2030 (2).

Global levels of food insecurity are expected to increase further, a trend already seen since 2014, particularly in the Caribbean and Latin America, adding to already high levels in Africa and South Asia (2). The world population is predicted to rise by a quarter by 2050 (8), with the population of Sub-Saharan Africa expected to double in the same timeframe, exacerbating prevalent undernutrition there. As well as a larger population, per capita meat demand is rising in many parts of the world (9). This is particularly prominent in regions of highest economic growth, rather than in low-income countries, with the relationship between affluence and meat consumption well-documented (9, 10). The combination of rising populations and affluence are intensifying food insecurity issues in low-income countries (9); identifying alternative, more sustainable protein sources is vital in combatting rising undernutrition and achieving sustainable food security.

Entomophagy, or the consumption of insects by humans, has substantial potential in combatting these issues and is widely practiced globally, particularly within traditional communities (11). Despite common misconceptions, communities practicing entomophagy widely regard insects as a delicacy rather than a “hardship” food in times of starvation (12); for instance, locusts are thought to have been popular among the social elite of the ancient Near East (13). It is becoming increasingly clear that entomophagy has played a pivotal role in providing nutrition throughout hominin evolution, with insect species being identified in human coprolites and some researchers even suggesting a homology between insectivory in human and non-human primates (14). A huge diversity of edible insect species are known (15–17), with over 2,000 species formally listed (18). The prevalence of entomophagy, as well as the species eaten, is highly geographically variable. For example, at least 250 species have been identified in diets in Africa across a diverse array of insect orders, the most speciose of which are Lepidoptera, Orthoptera, and Coleoptera (17, 19, 20). Similarly, at least 55 species have been identified in Japan, where Orthoptera and Hymenoptera are particularly popular (21), whilst 164 species have been identified across Lao People's Democratic Republic, Myanmar, Thailand, and Vietnam (22).

The benefits of edible insects in achieving sustainable food security are many. They are widely regarded to constitute a highly nutritious foodstuff. Although variable between insect species (23, 24), the high protein and fat content of edible insects compares favorably to meat and fish (12, 24–27). Furthermore, the amino acid profiles of several species have been demonstrated to contain a high proportion of essential amino acids; this was shown in *Imbrasia belina, Oryctes rhinoceros, Rhynchophorus phoenicis*, and *Macrotermes bellicosus*, all of which are popular in Africa (26), as well as in *Hermetia illucens* and *Musca domestica* (25, 28, 29). Several species of popular edible insects, including *R. phoenicis* and *M. bellicosus*, have been shown to compensate for certain limiting amino acids in cereal- and staple-based diets (23, 26). Furthermore, the fatty acid profiles of many edible insects compare favorably with meat and fish (23, 24). For example, Hussein et al. (29) identified a high content of unsaturated, particularly monounsaturated, fatty acids in *M. domestica* larvae. Similarly, Womeni et al. (27) found a high proportion of polyunsaturated fatty acids in *R. phoenicis, Homorocoryphus nitidulus*, and *Zonocerus variegates*, with particular representation of linoleic and α-linolenic acid, omega-6 and omega-3 fatty acids essential in development (24). This could compensate for the lacking representations of these essential fatty acids in staple vegetable oils (27). Edible insects also present a promising source of micronutrients (12). Iron and zinc deficiencies are prevalent and dangerous, particularly in developing countries (30, 31); entomophagy could provide a combative strategy, with iron and zinc levels shown to exceed that of beef in several edible insect species (24).

Not only are they nutritious but edible insects also provide a more efficient, cheaper, and less environmentally damaging nutrient source than vertebrate livestock species. As insects are cold-blooded invertebrates, they are highly efficient in converting input feed to edible matter (12, 15, 24); estimates suggest that around 25 kilograms of feed are required per kilogram of edible beef, yet this reduces to around two kilograms for edible insects (23, 32), thus insect rearing exerts far lower land and environmental pressure. Insects also emit far lower levels of greenhouse gases and ammonia than cattle and swine livestock (12, 23, 24, 33) and can be raised on organic waste substrates, recycling these in the process reducing feedstock demand relative to other agro-species. Using organic waste substrates can reduce waste volume and convert this into nutritious insect meal and useful co-products such as fertilizers and soil conditioners (34). Diener et al. (35) documented a 70% reduction in organic waste matter by *H. illucens* larvae, whilst Sheppard et al. (36) showed over a 50% reduction in manure waste, again with *H. illucens* larvae. Similar outcomes have been seen with other species, including *M. domestica* (23, 24).

The extensive benefits of edible insects and entomophagy cannot all be described here. Briefly, insect farming is also thought to pose far lower risk of agro-zoonosis emergence due to high human-insect taxonomic distance relative to vertebrate species (12, 23, 24). For example, one theory for the emergence of SARS-Cov-2 in humans is a cross-species transmission event at a meat market (37), our susceptibility to which may have been reduced were a greater emphasis put on agro-insect species. Furthermore, post-raising, edible insects can be feedstock to a variety of processes rather than being limited to human consumption; this includes medicines (38–41), biodiesel (42, 43), and feed for other agro-species, including poultry and fish (44–49). Finally, due to the small capital investment and expertise requirements of insect farming, and the high profit potential (50), insect rearing can provide livelihoods to the poorest individuals and communities (12). As a result of these clear benefits, there has been recent interest in the semi-cultivation and domestication of edible insects into mini-livestock (24, 51). This interest is emphasized by predictions that the edible insect market will value over six billion dollars by 2030 (52).

Despite these huge benefits in combatting undernutrition and promoting sustainable development, there has been a recent decrease in the prevalence of ento-ethno practices across traditional communities (53), where the benefits could be highest. This has generally been attributed to three main factors. Firstly, many communities are thought to be abandoning their traditions and are becoming increasingly westernized in their customs (53). DeFoliart (54) condemns western aversion to entomophagy and argues that it is a detriment to the prevalence of traditional uses of edible insects. Secondly, many argue that historical and recent edible insect over-exploitation has led to reduced supply for entomophagy (53). For example, Ramos-Elorduy (55) reports a decrease in the populations of several edible insect species in Mexico through over-exploitation. Finally, continued use of agricultural pesticides has been widely implicated in reducing edible insect populations and harvests (56, 57). Therefore, we must act quickly to conserve traditional knowledge of the prevalence, benefits, and potential of edible insect usage before it is lost. This includes documenting the traditional uses of edible insects in communities globally (53), assessing their nutritional and chemical value (12, 24, 53), and quantifying the extent to which they can meet the desired nutrition and sustainable development goals (53). There has also been a recent emphasis on research for promoting large-scale edible insect farming, including the innovation of mass-production technologies, development of international ento-ethno legislature, and education of new consumers (12, 24).

Recent strides have been made to nutritionally profile a variety of edible insect species and the number of entomophagy-related publications annually has increased from <10 pre-2012 to 114 in 2020. The general consensus is that edible insects are of high nutritional value and can potentially constitute an effective alternative feedstuff to meat and fish (12, 24, 58, 59). However, it is also widely accepted that edible insect nutrient profiles have major interspecific differences. For example, Rumpold and Schluter (59) identified a protein content range of 8.85–71.1% on a dry matter basis within Coleoptera. Similarly, high differences in protein, lipid, and ash, an indicator of mineral content (60), as well as in amino acid profiles, have been reported between *R. phoenicis, Z. variegatus*, and *M. bellicosus* (27, 61). Not only does interspecific variation exist but there is also within-species variation, including between different life stages, populations, and diets (23, 59, 62). *H. illucens* larvae reared on different substrates have been shown to diverge in nutrient profile, in particular in fatty acids, introducing the potential of modulating edible insect nutritional provision through substrate modification (63–65). More data is required to resolve this nutritional variation and fully elucidate the nutritional potential of insects (59). There are also growing concerns that some nutritional research is methodologically inconsistent and of low quality and yet is used to justify entomophagy's health benefits (66). Furthermore, the nutritional literature can often be taxonomically restricted and biased, with much emphasis on species of commercial or economic importance to major rearing companies rather than those of socio-economic or health benefit to traditional communities. For example, 25 times as many papers are found featuring “*Tenebrio* nutrition” than for “*Cirina* nutrition.” *Tenebrio* is a genus containing the yellow mealworm which is commercially raised for the pet food industry whereas *Cirina* is a genus of moth popular for consumption in Africa. Therefore, the nutritional literature must expand in order to maximize the health, social, livelihood, and sustainability benefits of entomophagy.

Here we analyzed proximate and fatty acid profiles of five species of edible insect of socio-economic importance in West Africa, a region with an emerging profile in ento-ethno research. These species, and the abbreviation used to refer to them in tables and figures, include *Hermetia illucens* (black soldier fly; BSF), *Musca domestica* (house fly; HSF), *Rhynchophorus phoenicis* (African palm weevil; PL), *Cirina butyrospermi* (shea tree caterpillar; STC), and *Macrotermes bellicosus* (African termite; TM). These nutritional profiles, which can be used in future research, are here combined with proximate, fatty acid, and also amino acid data present in the literature for these five species in order to rigorously assess interspecific nutrient differences. Through combining original and literature data, we provide an improved consensus of the nutritional offering of these five important species which have highest potential in combatting undernutrition and promoting sustainable development in Africa.

## Materials and Methods

### Nutritional Analyses

#### Sample Collection and Preparation

Larvae of *Hermetia illucens, Musca domestica, Rhynchophorus phoenicis, Cirina butyrospermi* and adults of *Macrotermes bellicosus* were obtained from the field in Ghana through sampling.

Samples were inactivated by putting them in 70% alcohol and dried by freezing in liquid nitrogen before being milled to flour.

The five insect meal flours were analyzed for proximate and fatty acid content at the Chemical Laboratory of the International Center for Insect Physiology and Ecology (*icipe*) in Nairobi, Kenya.

#### Proximate Analysis

Dried and milled *H. illucens, M. domestica, R. phoenicis*, and *C. butyrospermi* samples were assessed for proximate content using analytical procedures outlined by the Association of Official Analytical Chemists (67). Briefly, dry matter was assessed by oven drying method at 105°C for 24 h (67). Ash content, generally used as an indicator of the mineral content of foods (60), was determined by overnight incineration at 550°C, whilst organic matter was evaluated by subtracting this ash content from 100. Total nitrogen in the samples was assessed using the Kjeldahl method, with the crude protein content determined by multiplying total nitrogen by a conversion factor of 6.25. Crude fat content was evaluated using a Velp solvent extractor (SER 148/6; Velp Scientifica, Italy) with an ethyl ether extractant. Finally, neutral detergent fiber and acid detergent fiber were analyzed using a Velp fiber analyzer (FIWE 6; Velp Scientifica, Italy) and reagents described by Van Soest et al. (68).

All proximate measures, other than dry matter, were evaluated on a % dry matter basis; dry matter, organic matter, and ash content were determined in triplicate whilst all other proximate nutrients were in duplicate, except for *M. domestica* in which all proximate measures were evaluated in duplicate. All replication analyses were carried out using different batches of insect samples.

#### Fatty Acid Profiles

Fatty acid profiles were produced for each of the five species of edible insect according to the methods of Cheseto et al. (69) and Christie (70). Briefly, fatty acids were analyzed following conversion to their corresponding methyl esters; esterification was conducted by adding a solution of sodium methoxide in dry ethanol to each sample followed by extraction with GC-grade hexane. Fatty acid methyl esters were then analyzed by GC-MS using a 7950A gas chromatograph system with a 5975C mass selective detector (Agilent Technologies Inc., CA, USA) and HP-5 ms capillary column (length: 30 m, internal diameter: 0.25 mm, film thickness: 0.25 μm; Agilent Technologies Inc., CA, USA). An oven temperature programme of 35°C (5 min) increasing at 10°C per min to 285°C (20.4 min) was used, whilst a constant detector temperature of 230°C and 180°C for the ion source and quadrupole was conserved. The carrier gas was helium (1.25 ml per min flow rate). An acceleration energy of 70 eV was used to obtain electron impact mass spectra, with a 40–550 m/z mass range used for fragment ion analysis, having set a 3.3-min delay time for the filament. Fatty acid methyl ester identification was done by comparing fragmentation patterns and retention times to known fatty acid methyl ester standards where available and library-MS database reference spectra. Standard methyl octadecanoate of known concentrations (0.2–125 ng/μL) was subsequently analyzed under the same GC-MS conditions to obtain a linear calibration curve [y = 7E + 06x – 4E + 07 (*R*^2^ = 0.9757)] that was used for external quantification of fatty acid methyl esters.

Fatty acid profiles for each species were evaluated in triplicate, except for *M. domestica* and *M. bellicosus* which were both performed in duplicate, and are presented on a % total fatty acid basis.

### Nutritional Meta-Analysis

#### Literature Search String, Acceptance Criteria, and Data Extraction

In order to evaluate interspecific differences in proximate and fatty acid profiles the nutritional literature was rigorously searched and published nutritional profiles were combined with the profiles produced in this study. Although not evaluated originally for each species here, amino acid profiles were also extracted from the literature to assess interspecific differences. The following Google Scholar search string was employed to identify papers, published between 2000 and 2021, concerning the nutritive content of the five species analyzed here:

“*Nutrition AND Edible AND (Entomophagy OR Proximate OR “Fatty Acid” OR “Amino Acid”) (“Species Binomial Name”)—(“Literature Review”)—(“Meta-analysis”)”*

The resultant papers were then briefly read and subjected to the following inclusion criteria to identify relevant publications before being read in detail:

- The paper is an original article and has been through a peer review process, rather than being a review/meta-analysis, which uses external data, or a thesis/preprint.
- The paper concerns one or more of the relevant species (*H. illucens, M. domestica, R. phoenicis, C. butyrospermi*, or *M. bellicosus*).
- The paper specifically measures species nutritive content rather than addressing some other aspect of insect physiology, ecology, or evolution.

Due to the literature search being sorted by relevance to the search string and the high number of returned papers for certain species, with the BSF and HSF searches returning 1,380 and 1,060 papers, respectively, the search was terminated if 50 papers had elapsed since the last relevant one was identified. Relevant papers were then read in detail and subjected to the following further inclusion criteria before the data was extracted:

- The paper nutritionally profiles the desired life-stage of each species. For the species analyzed, the desired life-stages were as follows: larvae for *H. illucens, M. domestica, R. phoenicis*, and *C. butyrospermi*; adult for *M. bellicosus*.
- The paper nutritionally profiles the unadulterated insect sample, including no mixing with external ingredients, no cooking, and no defatting.
- The paper follows standard methods where possible, with no inappropriate sample preparation.
- The paper reports nutritional results in a form consistent with the wider literature and the nutritional analyses presented here. This included units of % dry matter, % total fatty acids, and g/100 g crude protein for proximate, fatty acid, and amino acid profiles, respectively.

For each paper satisfying all of the above criteria, proximate, fatty acid, and amino acid data was extracted for each species analyzed. The number of repeats used by each paper in producing their nutritional profiles was not accounted for during data extraction. Consequently, each paper, including the original profiles produced here, contributed a maximum of one datapoint to the final dataset for each of the proximate, fatty acid, and amino acid analyses conducted for each species analyzed. All papers used are given in Supplementary Table 1.

#### Manipulation of Literature Data

##### Fatty Acid Profiles

There is high heterogeneity in the nutritional literature in terms of the fatty acids identified and the fatty acid naming system used, with some papers opting for the trivial names whilst others prefer the systematic names or lipid numbers. Therefore, certain steps were taken to increase the comparability of the fatty acid literature.

Firstly, it was ensured that every paper contained the same fatty acids under a consistent naming system; where a paper did not identify a particular fatty acid, it was introduced at a content of 0%. Secondly, papers with a high proportion (>2.5%) of ambiguously named fatty acids, meaning that the specific isomer cannot be discerned, were removed. Where ambiguously named fatty acids accounted for <2.5% of a particular paper's fatty acid profile then the paper was retained but without the ambiguous fatty acid. Where a particular fatty acid was not reported in any form other than the ambiguous form across the utilized literature then the ambiguous name was retained; this was the case for pentadecenoic acid, docosadienoic acid, and decenoic acid. Furthermore, singleton fatty acids, those only identified in a single publication, were removed to reduce the impact of experimental variability and because their inclusion would increase the fatty acid diversity of more represented species. The sum of fatty acid profile in each paper was fixed to 100% by introducing an “Others” category when the profile was below 100%, for instance due to the removal of ambiguous fatty acids. Finally, where a paper reported *M. domestica, R. phoenicis, C. butyrospermi*, or *M. bellicosus* fatty acid profiles in differing units, they were converted to the standard units of % total fatty acids due to *H. illucens* being over-represented in the literature.

This manipulation left a final fatty acid dataset comprised of 16 *H. illucens*, six *M. domestica*, nine *R. phoenicis*, three *C. butyrospermi*, and five *M. bellicosus* papers.

##### Amino Acid Profiles

There is high heterogeneity in the units used to report amino acid profiles in the literature, with amino acid content in g/100 g crude protein, % dry matter, and % total amino acids all being present. Units of g/100 g crude protein were chosen due to being the most prevalent. As a result of *H. illucens* being very heavily represented in the edible insect literature relative to the other species, where a paper reported *M. domestica, R. phoenicis, C. butyrospermi*, or *M. bellicosus* amino acid profiles on a % dry matter basis, this was converted to the selected units of g/100 g crude protein using the paper's reported crude protein value. This process left a final amino acid dataset comprised of six *H. illucens*, two *M. domestica*, eight *R. phoenicis*, two *C. butyrospermi*, and four *M. bellicosus* papers. Unlike for proximate and fatty acid analyses, this dataset was comprised entirely of profiles derived from the literature as original amino acid profiles were not produced here.

#### Statistical Analyses

All statistical analyses were conducted in R (v4.1.0) (71) with RStudio (v1.4.1106) (72).

##### Proximate Content

Proximate content differences between the five analyzed species were assessed by a series of non-parametric Kruskal-Wallis tests, with *post-hoc* differences evaluated using the package, dunn.test (73). Visualizations were produced with the package ggplot2 (74). This was performed for the three macronutrients most widely reported in the literature, which included ash, crude protein, and crude fat; other proximate nutrients were not reported frequently and consistently enough to allow statistical evaluation of interspecific differences.

##### Fatty Acid Profiles

Fatty acid profile structuring between species was assessed by principle component analysis. The PCA output was visualized using the package ggbiplot and interspecific differences in the distributions of the two primary principle components were evaluated using non-parametric Kruskal-Wallis tests, with *post-hoc* differences evaluated using the package, dunn.test (73). Furthermore, reported fatty acids were classified based on saturation status and, for each paper, the sum of saturated fatty acids, mono-unsaturated fatty acids, and poly-unsaturated fatty acids was determined. Interspecific differences in the degree of saturation were assessed by a series of non-parametric Kruskal-Wallis tests, with *post-hoc* differences again being evaluated with the package, dunn.test (73). The association of each saturation class with each of the two primary principle components in the fatty acid analysis was also assessed by a correlation test using the Pearson method.

##### Amino Acid Profiles

There is high variation in the literature in terms of which amino acids are profiled, with some papers reporting only essential amino acids, others reporting all amino acids, whilst others report some other subset of amino acids. Consequently, it was not possible to statistically evaluate how amino acid profiles structure across species. Instead, the mean content of each essential amino acid was determined for each species and these, along with the essential amino acid requirements for adults (75), were visualized in ggplot2 (74) in order to assess to what extent these species' amino acid profiles meet human requirements.

## Results

### Nutritional Analyses

#### Proximate

The insect species generally contained high protein levels, ranging from 31% in *R. phoenicis* to 64% in *C. butyrospermi*, demonstrating their potential as a protein source, with *C. butyrospermi* and *M. domestica* even exceeding beef in their protein content. The highest fat content was found in *R. phoenicis* (65%), greatly exceeding that of the other insect species and of beef (41%). Besides *R. phoenicis*, the insect species were generally characterized by low fat levels (18.03%, 11.49%, and 12.18% in *H. illucens, M. domestica*, and *C. butyrospermi*, respectively). Ash content was highest in the dipteran species (17.71% and 9.84% in *H. illucens* and *M. domestica*, respectively), whilst levels of neutral and acid detergent fiber were similar in *C. butyrospermi* and *M. domestica* but lower in *H. illucens* (Table 1).

**Table 1:** Original proximate profiles produced in this study for the four analyzed species of edible insects alongside a beef control, obtained from van Huis et al. (24).

	**BSF**	HSF^*^	**PL**	**STC**	Beef^**^
Dry Matter (*n* = 3)	92.24 (±0.00)	88.45 (±0.00)	39.25 (±0.00)	87.45 (±0.00)	–
Organic matter (*n* = 3)	82.29 (±0.42)	90.12 (±0.28)	98.62 (±0.17)	93.59 (±0.44)	–
Ash (*n* = 3)	17.71 (±0.42)	9.84 (±0.28)	1.38 (±0.17)	6.41 (±0.44)	–
Crude protein (*n* = 2)	44.82 (±0.40)	61.00 (±0.66)	31.05 (±0.55)	63.64 (±0.22)	55.00
Crude fat (*n* = 2)	18.03 (±0.13)	11.49 (±0.46)	65.35 (±0.14)	12.18 (±0.09)	41.00
NDF (*n* = 2)	39.94 (±0.71)	54.89 (±1.10)	–	56.22 (±0.00)	–
ADF (*n* = 2)	15.57 (±0.37)	37.15 (±0.54)	–	32.45 (±0.31)	–

*All measures, other than dry matter, are presented as a % of dry matter*.

*BSF, black soldier fly (Hermetia illucens); HSF, house fly (Musca domestica); PL, African palm weevil (Rhynchophorus phoenicis); STC, shea tree caterpillar (Cirina butyrospermi)*.

Insect dry matter, organic matter, and ash values are presented as mean ± SE of triplicate measures whilst crude protein, crude fat, neutral detergent fiber (NDF), and acid detergent fiber (ADF) are mean ± SE of duplicates, other than for HSF(^*^) where all proximate measures are in duplicate.

** *Crude protein and fat content of beef from van Huis et al. (24)*.

#### Fatty Acid Profiles

The black soldier fly, *H. illucens*, contained the highest proportion of saturated fatty acids (61.36%), whilst the other dipteran, *M. domestica*, had the lowest (44.74%) (Table 2). In *H. illucens*, these saturated fatty acids were primarily composed of dodecanoic acid (8.37%), which was absent or at low level in the other species, tetradecanoic acid (5.01%), hexadecanoic acid (9.44%), and octadecanoic acid (6.61%), which were also prevalent in the other species, with *M. bellicosus* containing 24.26% hexadecanoic acid.

**Table 2:** Fatty acid profiles of the five analyzed species of edible insect.

**Fatty acid (%)**			**BSF** **(***n*** = 3)**	**HSF** **(***n*** = 2)**	**PL** **(***n*** = 3)**	**STC** **(***n*** = 3)**	**TM** **(***n*** = 2)**
**Systematic name**	**Trivial name**	**Lipid No**.					
2-methyl propanoic acid	–	C4:0i	0.00	0.42	0.00	0.00	0.00
2-methyl butanoic acid	–	C5:0a	1.15	0.00	0.00	0.00	0.00
Hexanoic acid	Caproic acid	C6:0	2.61	0.00	0.00	0.64	0.00
Octanoic acid	Caprylic acid	C8:0	0.00	0.00	0.00	1.08	0.00
Non-anoic acid	Pelargonic acid	C9:0	0.52	0.00	0.00	0.00	0.00
Decanoic acid	Capric acid	C10:0	0.62	0.00	0.00	0.00	0.00
10-methyl undecanoic acid	–	C12:0i	0.00	1.86	0.59	0.00	0.00
Dodecanoic acid	Lauric acid	C12:0	8.37	1.23	1.57	0.96	0.00
10-methyl dodecanoic acid	–	C13:0a	0.00	0.53	0.00	0.00	0.00
Tridecanoic acid	Tridecylic acid	C13:0	0.95	2.48	0.00	0.00	0.00
Tetradecanoic acid	Myristic acid	C14:0	5.01	4.80	4.66	2.89	8.98
9-methyl tetradecanoic acid	–	–	1.00	1.04	0.00	0.00	0.00
12-methyl tetradecanoic acid	–	C15:0a	1.30	0.00	0.00	0.00	0.00
13-methyl tetradecanoic acid	–	C15:0i	3.46	2.12	0.00	0.67	0.00
Pentadecanoic acid	Pentadecylic acid	C15:0	2.31	1.93	0.81	2.67	0.00
3-methyl pentadecanoic acid	–	–	0.85	0.74	0.00	0.00	0.00
Hexadecanoic acid	Palmitic acid	C16:0	9.44	9.63	27.13	14.95	24.26
10-methyl hexadecanoic acid	–	–	0.00	0.75	0.00	0.00	0.00
14-methyl hexadecanoic acid	–	C17:0a	3.20	0.00	0.70	0.00	0.00
15-methyl hexadecanoic acid	–	C17:0i	1.02	1.92	0.93	0.00	0.00
Heptadecanoic acid	Margaric acid	C17:0	1.06	0.00	1.06	2.91	0.00
Octadecanoic acid	Stearic acid	C18:0	6.61	6.51	7.88	18.28	15.00
17-methyl octadecanoic acid	–	C19:0i	1.21	0.00	0.89	0.00	0.00
Non-adecanoic acid	Non-adecylic acid	C19:0	1.99	0.80	1.46	2.78	0.00
18-methyl non-adecanoic acid	–	C20:0i	1.02	0.00	0.90	1.94	5.35
Icosanoic acid	Arachidic acid	C20:0	2.60	3.10	3.18	1.96	5.33
20-methyl heneicosanoic acid	–	C22:0i	3.82	0.00	2.31	0.00	0.00
Docosanoic acid	Behenic acid	C22:0	0.00	3.03	0.00	2.76	0.00
Tetracosanoic acid	Lignoceric acid	C24:0	1.27	0.00	0.00	1.41	0.00
Non-ahexacontanoic acid	–	C69:0	0.00	1.84	0.00	0.00	0.00
5-dodecenoic acid	Lauroleinic acid	C12:1n-7	0.00	0.00	0.00	2.42	0.00
9-tetradecenoic acid	Myristoleic acid	C14:1n-5	0.82	0.59	0.00	0.00	0.00
11-tetradecenoic acid	–	C14:1n-3	0.00	0.94	0.00	0.00	0.00
7-hexadecenoic acid	Hypogeic acid	C16:1n-9	0.99	0.00	0.00	0.84	0.00
9-hexadecenoic acid	Palmitoleic acid	C16:1n-7	5.03	6.74	5.89	4.26	0.00
11-hexadecenoic acid	Lycopodic acid	C16:1n-5	0.00	2.24	0.00	0.00	0.00
8-heptadecenoic acid	Civetic acid	C17:1n-9	0.00	0.00	0.94	0.00	0.00
9-heptadecenoic acid	Margaroleic acid	C17:1n-8	0.00	0.00	0.66	1.37	0.00
10-heptadecenoic acid	–	C17:1n-7	1.14	0.89	0.90	2.42	0.00
6-octadecenoic acid	Petrolselinic acid	C18:1n-12	0.00	0.00	2.02	1.12	7.97
8-octadecenoic acid	–	C18:1n-10	0.00	3.60	0.00	0.00	0.00
9E-octadecenoic acid	Elaidic acid	C18:1n-9	7.91	5.09	0.00	0.00	12.48
9-octadecenoic acid	Oleic acid	C18:1n-9	2.59	2.24	0.00	0.00	4.41
11-octadecenoic acid	Asclepic acid	C18:1n-7	4.07	4.29	29.38	0.71	0.00
13-octadecenoic acid	–	C18:1n-5	0.00	1.50	0.00	0.89	5.93
11-icosenoic acid	Gondoic acid	C20:1n-9	0.97	1.34	0.63	0.00	0.00
13-icosenoic acid	Paullinic acid	C20:1n-7	2.56	0.00	1.36	0.00	0.00
7,10-hexadecadienoic acid	–	C16:2n-6	0.79	2.52	0.00	0.00	0.00
9,12-octadecadienoic acid	Linoleic acid	C18:2n-6	6.43	8.84	2.20	5.72	8.16
6,9,12-octadecatrienoic acid	γ-linolenic acid	C18:3n-6	0.94	0.81	0.00	1.41	0.00
5,8,11,14-icosatetraenoic acid	Arachidonic acid	C20:4n-6	0.00	1.00	0.00	0.00	0.00
9,12,15-octadecatrienoic acid	α-linolenic acid	C18:3n-3	0.00	0.00	0.00	17.12	0.00
11,14,17-icosatrienoic acid	–	C20:3n-3	0.00	0.00	0.00	2.93	0.00
5,8,11,14,17-icosapentaenoic acid	Timnodonic acid	C20:5n-3	0.00	2.94	0.00	0.00	0.00
9Z,11E-octadecadienoic acid	Rumenic acid	C18:2n-7	1.02	0.00	0.00	0.00	0.00
6Z,9Z,11E-octadecatrienoic acid	–	C18:3n-7	0.00	0.00	0.00	1.75	0.00
11,14-octadecadienoic acid	–	C18:2n-4	0.00	0.00	0.59	0.00	0.00
Others	–	–	3.40	9.69	1.36	1.14	2.11
ΣSFA	–	–	61.36	44.74	54.07	55.90	58.94
ΣMUFA	–	–	26.06	29.46	41.78	14.02	30.80
ΣPUFA	–	–	9.18	16.11	2.79	28.94	8.16
PUFA (n-3)			0.00	2.94	0.00	20.06	0.00
PUFA (n-6)			8.16	13.17	2.20	7.13	8.16

*Fatty acid content is reported as a % of total fatty acids*.

*BSF, black soldier fly (Hermetia illucens); HSF, House fly (Musca domestica); PL, African palm weevil (Rhynchophorus phoenicis); STC, shea tree caterpillar (Cirina butyrospermi); TM, African termite (Macrotermes bellicosus)*.

*SFA, saturated fatty acids; MUFA, mono-unsaturated fatty acids; PUFA, poly-unsaturated fatty acids*.

*Fatty acid profiles are presented as the mean of triplicate evaluations, other than the TM and HSF which were determined in duplicate*.

The palm weevil, *R. phoenicis*, had the highest mono-unsaturated fatty acid content (41.78%) and *C. butyrospermi* the lowest (14.02%). In *R. phoenicis*, this portion was primarily composed of 11-octadecenoic acid (29.38%), whilst oleic acid (9-octadecenoic acid) was absent; similarly, oleic acid was not detected in *C. butyrospermi* but was present in the other three species.

Contrary to mono-unsaturated content, the highest proportion of poly-unsaturated fatty acids was found in the caterpillar, *C. butyrospermi*, and the lowest in *R. phoenicis* (28.94 and 2.79%, respectively). Across the species, this portion was primarily comprised of n-6 fatty acids, with n-3 fatty acids being absent completely in *H. illucens, M. domestica, and M. bellicosus*. However, the opposite was found in *C. butyrospermi*, with n-3 fatty acids being more prevalent due to the very high α-linolenic acid content, which was not detected in the other species.

### Nutritional Meta-Analysis

#### Proximate Content

**Figure 1:**
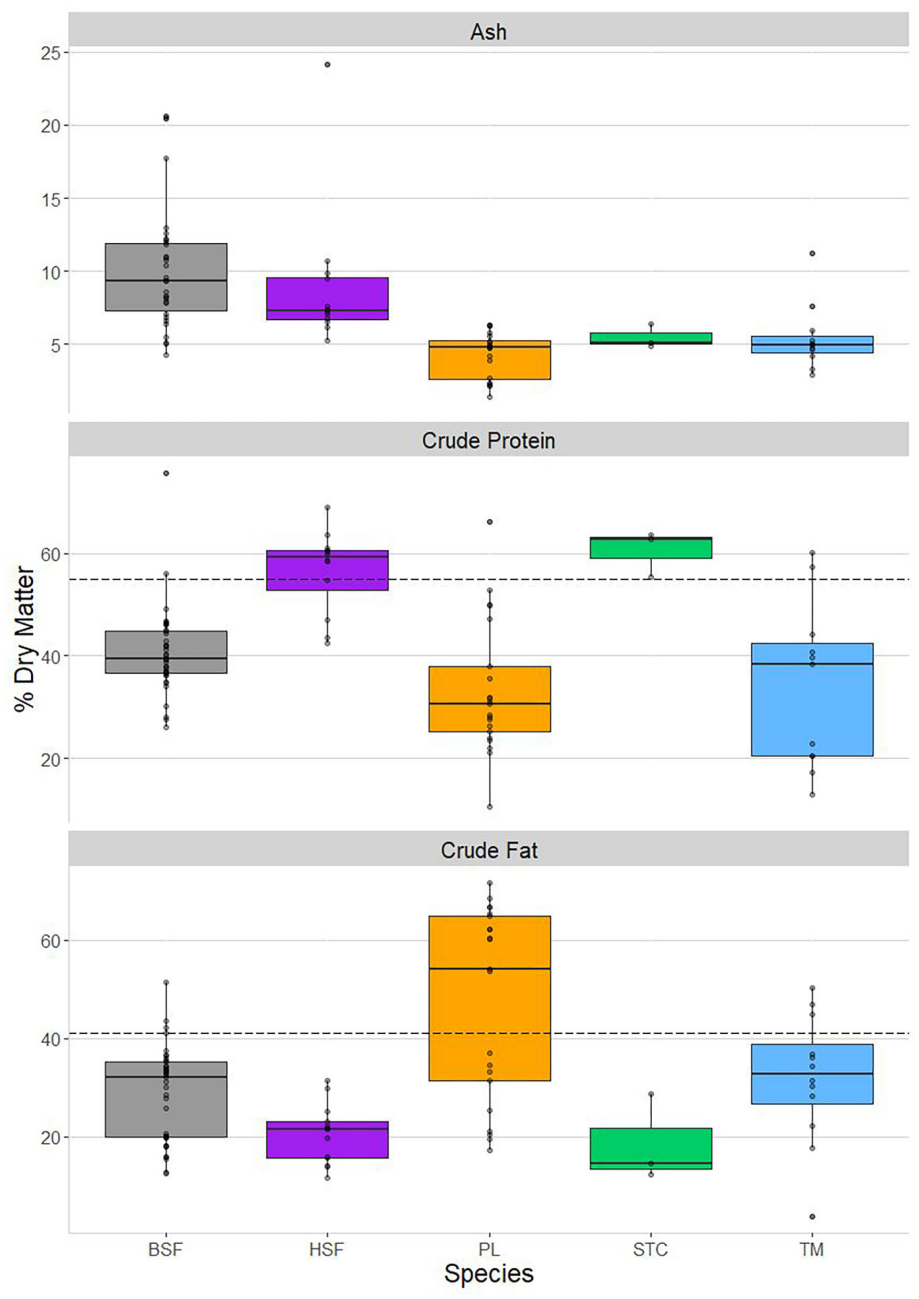
Proximate content, on a % dry matter basis, using both data analyzed in this paper and literature-sourced data. From top to bottom: ash, crude protein, and crude fat content. From left to right: *Hermetia illucens* (black soldier fly, BSF; in gray), *Musca domestica* (house fly, HSF; in purple), *Rhynchophorus phoenicis* (palm weevil, PL; in orange), *Cirina butyrospermi* (shea tree caterpillar, STC; in green), and *Macrotermes bellicosus* (termite, TM; in blue). The horizontal hashed lines in the bottom two panels represent the crude protein and crude fat content of beef, respectively, obtained from van Huis et al. (24).

Species differences in their ash, crude protein, and crude fat contents are shown in Figure 1, as well as how they compare with beef in the latter two nutrients. These three macronutrients were the only proximate measures of high enough sample size to allow rigorous inspection of interspecific differences after combining the proximate analyses conducted in this paper with the nutritional literature.

Highly significant interspecies differences were found in ash, crude protein, and crude fat content (Table 3). As was the case for the proximate profiles produced in this paper (Table 1), ash content was highest in *H. illucens* and *M. domestica*, which did not differ from each other but both differed significantly from the other three species, except when comparing *M. domestica* and *C. butyrospermi*. Ash accounted for around 5% of dry matter in the remaining three species, with no differences between them.

**Table 3:** Statistical output of a series of non-parametric tests: firstly, Kruskal-Wallis output, in the form *χ*^2^ (*p*-value), assessing whether species differ in their content of ash, crude protein, and crude fat, and below the post-hoc significance output determining whether individual species pairs differ in their levels of these proximate nutrients.

**Comparison**		**Ash**	**Crude protein**	**Crude fat**
Species		41.49 (*P* < 0.001)	30.71 (*p* < 0.001)	24.90 (*p* < 0.001)
BSF -	HSF	n.s.	***	**
	PL	***	^*^	**
	STC	^*^	**	n.s.
	TM	***	n.s.	n.s.
HSF-	PL	***	***	***
	STC	n.s.	n.s.	n.s.
	TM	**	***	**
PL -	STC	n.s.	***	**
	TM	n.s.	n.s.	n.s.
STC -	TM	n.s.	**	^*^

Significance level: p > 0.05 (n.s.) ^*^p < 0.05,

** *p < 0.01*,

*** *p < 0.001*.

*BSF, Black soldier fly (Hermetia illucens); HSF, House fly (Musca domestica); PL, Palm weevil (Rhynchophorus phoenicis); STC, Shea tree caterpillar (Cirina butyrospermi); TM, African termite (Macrotermes bellicosus)*.

Crude protein was found to be most prevalent in *C. butyrospermi* and *M. domestica* at around 60%, corroborating the patterns detected previously (Table 1). Although these species' protein content did not differ from each other, they were both significantly greater than the levels found in the remaining three insect species and both exceeded that found in beef (55%).

Crude fat was most prevalent in *R. phoenicis* and *M. bellicosus* and was lowest in *C. butyrospermi* and *M. domestica*, with significant differences existing between but not within these species-pairs. Furthermore, *R. phoenicis* was found to be highly variable in crude fat content, ranging from around 20 to over 70% of dry matter; this variable distribution can be seen to fit into two distinct clusters of datapoints, one at around 60–70% and the other below 40%.

#### Fatty Acid Content

**Figure 2:**
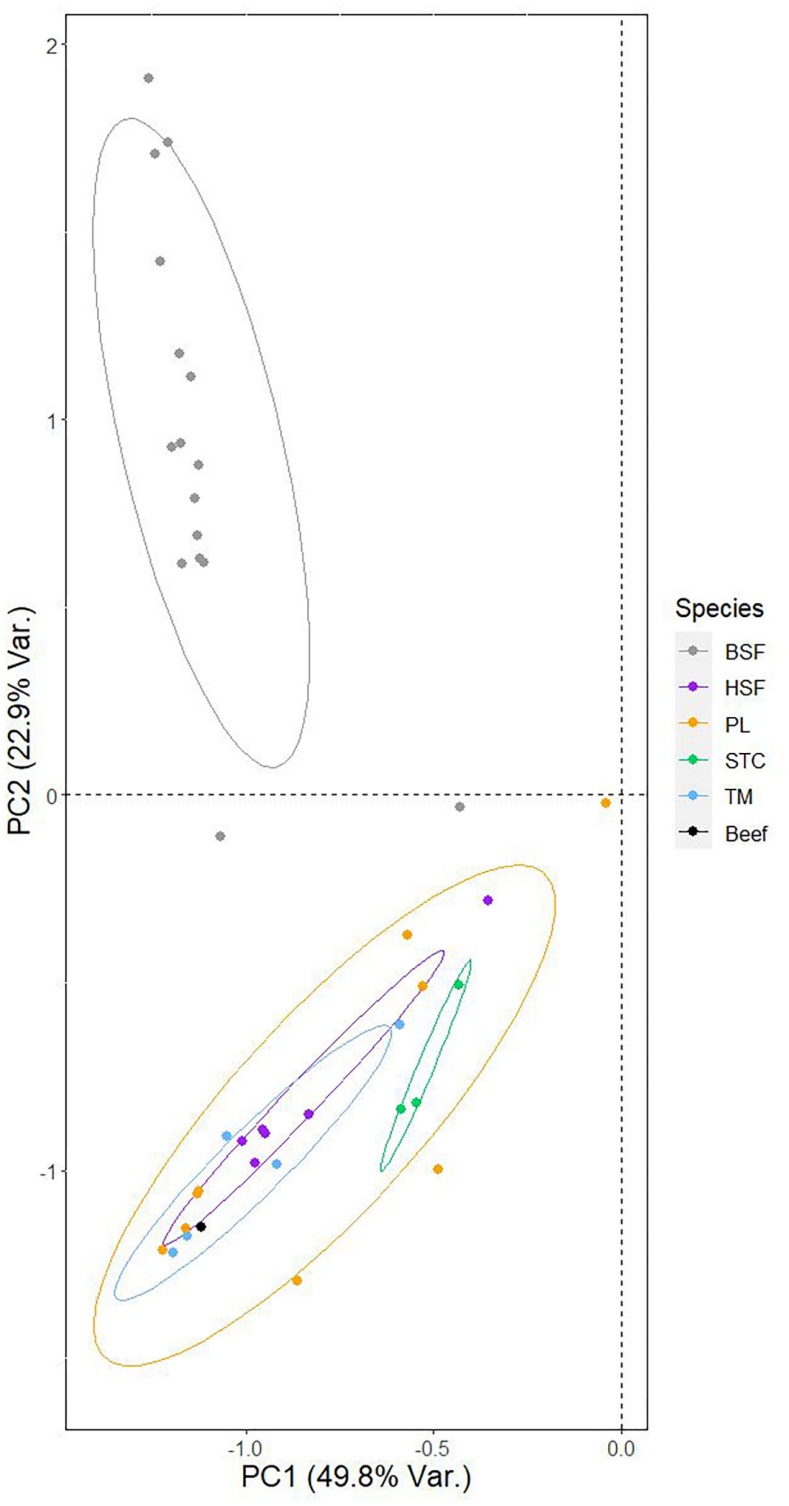
Principal component analysis of the fatty acid profiles of the five analyzed species of edible insect. The relationship between the two primary principal components and individual datapoints, corresponding to a fatty acid profile of an individual species presented either in the literature or analyzed in this study, is shown. Points are colored based on the species to which they belong, as are the ellipses surrounding each species' datapoints. Superimposed onto the plot is the position of the fatty acid profile of beef, obtained from van Huis et al. (24), relative to the two primary principle components.

The principal component analysis assessing between-species structuring in fatty acid profiles indicates clear, major differences between species in their position relative to PC1 and PC2, particularly in the case of PC2 (Figure 2, Table 4). Figure 2 also illustrates the relative position of the fatty acid profile of beef (24). In particular, the *H. illucens* data was found to diverge, forming a distinct cluster in the PCA while differences between the other species, and beef, were minimal.

**Table 4:** Statistical summary of non-parametric tests assessing how these species of edible insect differ in their fatty acid profiles.

		**PC1**	**PC2**	**∑SFA**	**∑MUFA**	**∑PUFA**	**∑PUFAn-6**	**∑PUFAn-3**
P(Variance)		49.76	22.88	–	–	–	–	–
Species		14.62 (**)	28.45 (***)	21.12 (***)	20.29 (***)	10.04 (*)	5.68 (n.s.)	9.15 (n.s.)
BSF -	HSF	**	***	***	**	n.s.	n.s.	n.s.
	PL	**	***	**	***	n.s.	n.s.	n.s.
	STC	**	*	n.s.	n.s.	**	n.s.	**
	TM	n.s.	***	*	*	n.s.	n.s.	n.s.
HSF-	PL	n.s.	n.s.	n.s.	n.s.	n.s.	*	n.s.
	STC	n.s.	n.s.	*	***	n.s.	n.s.	**
	TM	n.s.	n.s.	*	n.s.	n.s.	n.s.	n.s.
PL -	STC	n.s.	n.s.	n.s.	***	**	n.s.	**
	TM	n.s.	n.s.	n.s.	n.s.	n.s.	n.s.	n.s.
STC -	TM	*	n.s.	n.s.	*	*	n.s.	**
∑SFA	−0.27 (n.s.)	0.59 (***)	–	–	–	–	–	–
∑MUFA	0.09 (n.s.)	−0.53 (***)	–	–	–	–	–	–
∑PUFA	0.16 (n.s.)	−0.16 (n.s.)	–	–	–	–	–	–
∑PUFAn-6	−0.07 (n.s.)	−0.07 (n.s.)	–	–	–	–	–	–
∑PUFAn-3	0.33 (*)	−0.16 (n.s.)	–	–	–	–	–	–

*From top to bottom: the proportion of variance in the fatty acid dataset captured by each of the two major principle components in the PCA; Kruskal-Wallis output, in the form χ^2^ (significance level), assessing how species differ in their distribution of PC1 and PC2, as well as in their content of each fatty acid saturation class; significance levels of the post-hoc output of the Kruskal-Wallis test, showing which species pairs vary in their principle component distributions and in the degree of saturation of their fatty acid profiles; output of a series of correlation tests, using the Pearson method, assessing the relationship between each fatty acid saturation class and each of the two major principle components in order to provide biological context to the PCA output*.

Significance level: p > 0.05 (n.s.) ^*^p < 0.05 ^**^p < 0.01 p < 0.001^***^.

*BSF, Black soldier fly (Hermetia illucens); HSF, House fly (Musca domestica); PL, Palm weevil (Rhynchophorus phoenicis); STC, Shea tree caterpillar (Cirina butyrospermi); TM, African termite (Macrotermes bellicosus)*.

*SFA, saturated fatty acids; MUFA, mono-unsaturated fatty acids; PUFA, poly-unsaturated fatty acids*.

**Figure 3:**
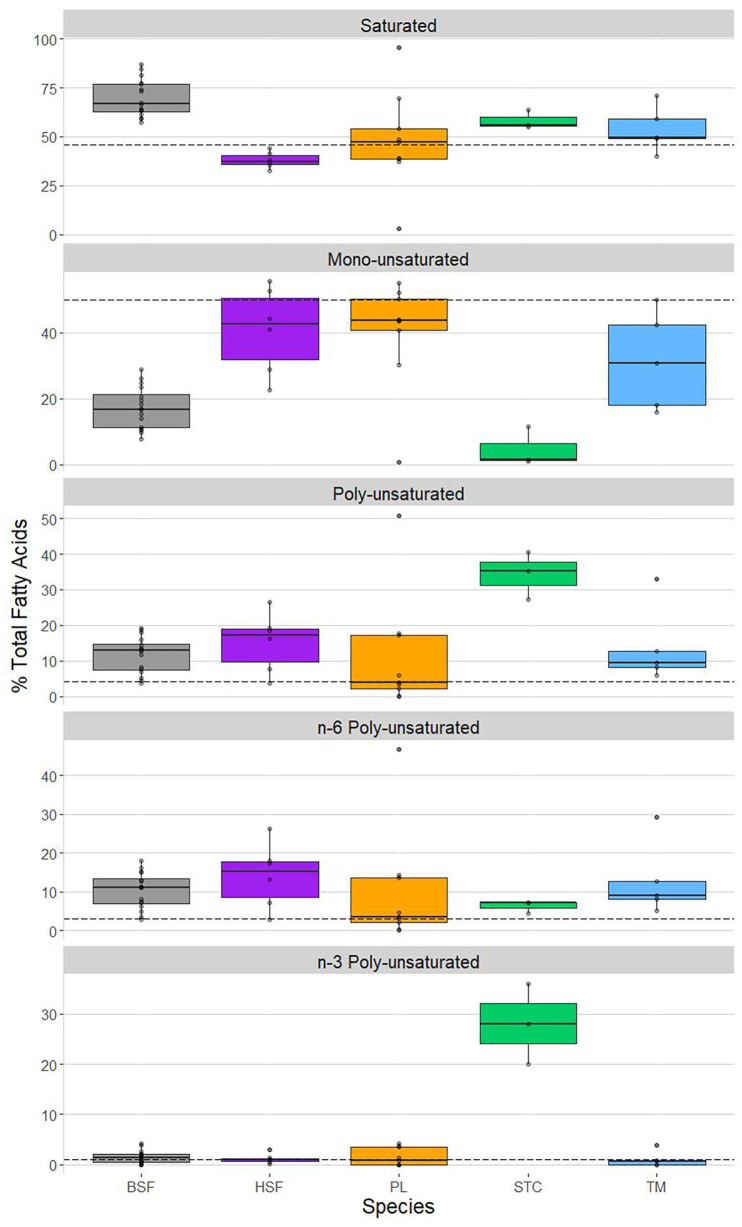
Variation in fatty acid profile saturation as a function of insect species. Shown are each species' content, as a % of total fatty acids, of, from top to bottom, saturated fatty acids, mono-unsaturated fatty acids, poly-unsaturated fatty acids, n-6 poly-unsaturated fatty acids, and n-3 poly-unsaturated fatty acids. Shown, from left to right, in each panel: *Hermetia illucens* (black soldier fly, BSF; in gray), *Musca domestica* (house fly, HSF; in purple), *Rhynchophorus phoenicis* (palm weevil, PL; in orange), *Cirina butyrospermi* (shea tree caterpillar, STC; in green), and *Macrotermes bellicosus* (termite, TM; in blue). The hashed horizontal lines in each panel represent the degree of saturation of the fatty acid profile of beef, obtained from van Huis et al. (24).

PC2 was shown to share a strong positive correlation with saturated fatty acid content and a strong negative correlation with mono-unsaturated fatty acid content (Table 4), suggesting these parameters may show the strongest interspecific structuring (Figure 3). *Hermetia illucens* showed the highest content of saturated fatty acids (~65%), corroborating the PCA output, higher than all compared species other than *C. butyrospermi* and exceeding that of beef. The lowest proportion of saturated fatty acids was found in *M. domestica*. Furthermore, mono-unsaturated fatty acid content was lowest in *H. illucens* and *C. butyrospermi* and highest in the other three species, with strongly significant differences existing between but not within these high and low groups.

Poly-unsaturated fatty acid content was highest in *C. butyrospermi*, exceeding that of the other four insect species and beef. This can primarily be attributed to n-3 fatty acids, of which *C. butyrospermi* contained around 30%, differing from the basal levels of the other species. Conversely, little interspecies variation was found in the content of n-6 fatty acids.

#### Amino Acid Content

**Figure 4:**
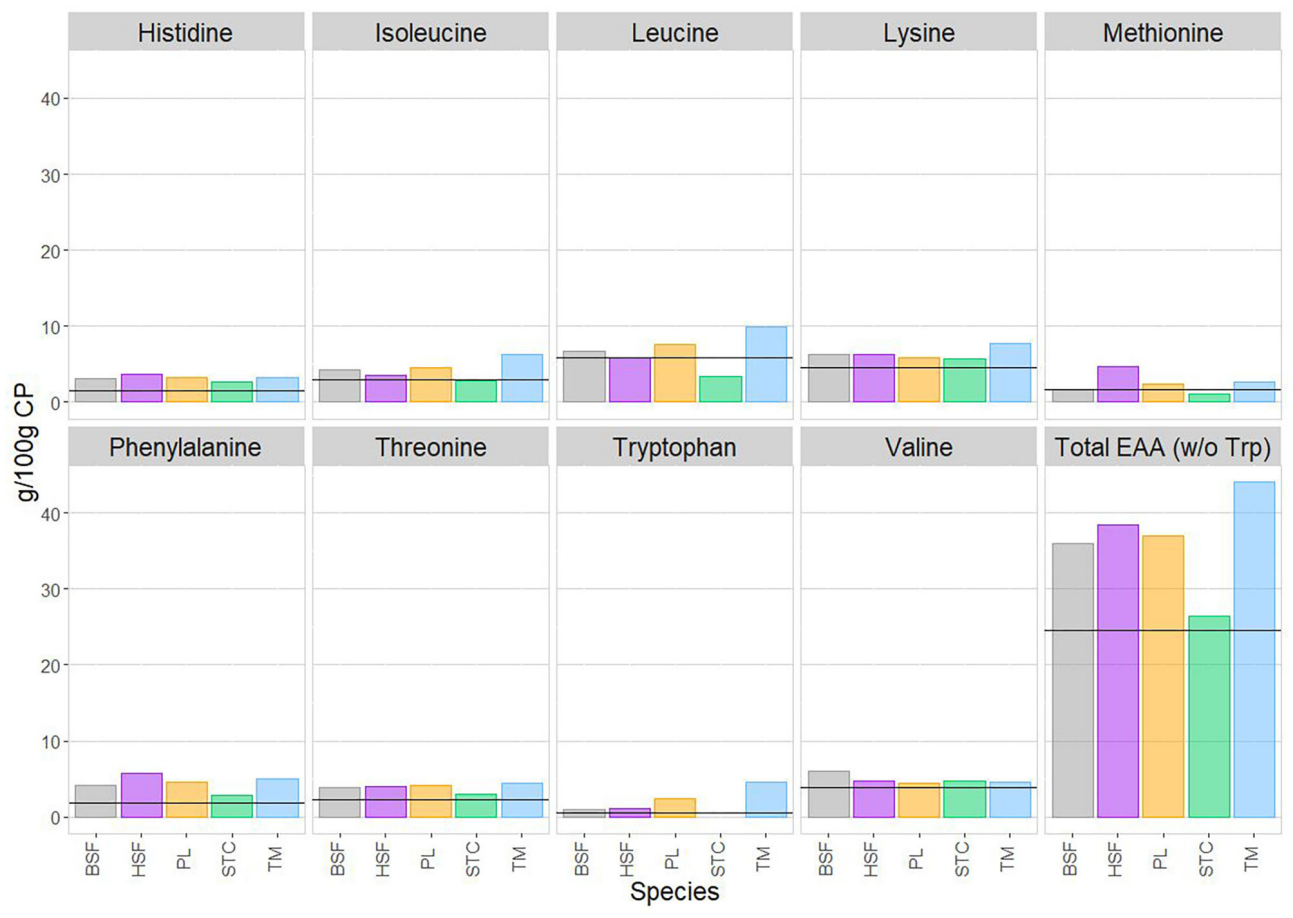
The mean content of each essential amino acid, in g/100 g of crude protein, in each species of edible insect, alongside the total sum of essential amino acids. As neither of the two published papers on *C. butyrospermi's* amino acid content reported tryptophan, the mean total essential amino acid contents have here been calculated in the absence of tryptophan. The horizontal lines superimposed onto each panel refer to current estimates for adult requirements of each amino acid, obtained from WHO/FAO/UNU (75). Within each amino acid panel, reported is, from left to right: *Hermetia illucens* (black soldier fly, BSF; in gray), *Musca domestica* (house fly, HSF; in purple), *Rhynchophorus phoenicis* (palm weevil, PL; in orange), *Cirina butyrospermi* (shea tree caterpillar, STC; in green), and *Macrotermes bellicosus* (termite, TM; in blue).

For each species, each essential amino acid is reported as the mean of data reported in the literature and in g/100 g of crude protein. No literature was found reporting the tryptophan content of *C. butyrospermi*, indicating the underrepresentation of this species in the nutritional literature, so the total sum of essential amino acids was evaluated in the absence of tryptophan. Also shown in Figure 4 are the concentrations of each amino acid required by the human adult (75).

For many amino acids, relatively little interspecies variation was detected (Figure 4). This was the case for histidine, lysine, phenylalanine, threonine, and valine, with all species showing concentrations of these amino acids exceeding that of human requirements.

Greater variation was detected in leucine and methionine, with the highest levels of these found in *M. bellicosus* and *M. domestica*, respectively. In both cases, levels in *C. butyrospermi* fell slightly short of human requirements. However, the small sample size of the *C. butyrospermi* amino acid data should also be noted, with only two papers contributing profiles.

For all other amino acids, all species exceeded human requirements, as did the total essential amino acid content of all species, demonstrating their nutritive potential.

## Discussion

Here, original nutritional profiles, including proximate and fatty acid data, have been produced for five edible insect species of socio-economic importance in West Africa, a region where entomophagy is highly popular (76) but has been relatively neglected in ento-ethno research. These profiles can be used in future research, adding to the available data, which has thus-far been lacking for some species; for example, the shea tree caterpillar, *C. butyrospermi*, evaluated in this study, has been neglected in the nutritional literature despite being popularly consumed. These original profiles have been combined here with those proximate, fatty acid, and also amino acid profiles previously produced in the literature in order to highlight interspecific variation in nutrient content. This has allowed rigorous assessment not only of whether these species have potential in combatting widespread undernutrition but also which of the five species show nutrient profiles most favorable for consumption.

### Nutritional Potential and Interspecific Variation

These results promote the general suitability and substantial potential of edible insects as a nutrient source. The ash and crude protein content values fit well within the range previously identified for edible insects, with ash previously reported at <1%-25.95% and protein at 4.9–77.13% (59) and 20–70% (77). Similarly, crude fat content, which here ranged from around 15% of dry matter in *C. butyrospermi* to around 55% in *R. phoenicis*, was broadly corroborated by the nutritional literature; Rumpold and Schlüter (59) reported a range of <1–77% across edible insects whilst this narrowed to 10–50% according to Xiaoming et al. (77). The insects analyzed here compare well with the protein and fat content of around 55 and 40%, respectively, previously reported for beef (24).

The composition of the crude fat portion, described in part by the fatty acid profile, was also shown here to be generally favorable in the insects. In particular, the insects showed a higher proportion of poly-unsaturated fatty acids than beef, which are recognized as essential for development in children and are often limited in landlocked countries with limited fish access (24). The amino acid profiles of these edible insect species generally exceeded the essential amino acid requirements of adult humans, with minor exceptions, such as the limited leucine and methionine content of the caterpillar.

These data confirm previous work that edible insects generally represent a viable and thus-far underutilized nutrient source, comparing extremely well to human requirements and traditional meat sources, such as beef (12, 24, 58, 59). Not only are they nutritionally comparable and often more desirable than beef but edible insects also provide numerous other benefits for sustainable food security, including the efficiency, low cost, and environmental benefits of raising them (23). However, the results presented here, as well as highlighting the potential of entomophagy, do promote certain insect species over others.

Much interspecific nutritional variation was found between the five species analyzed here, with implications for their utility in assuring sustainable food security. The protein content and quality of ingredients has been described as the most important consideration in food and feed production (29). Proteins have a wide variety of bodily functions, including providing the structural components of many tissues (26), promoting growth and recovery (78), being involved in biochemical processes (79), and maintaining immune function (80). Consequently, protein deficiency, which is prevalent globally (2, 81), threatens these processes. Therefore, our results indicate that the caterpillar, *C. butyrospermi*, and house fly, *M. domestica*, may be most suitable for human consumption, both showing the highest crude protein content and greatly exceeding that of the other three species.

Furthermore, ash is generally used as an indicator of the mineral content of foods (60), suggesting that *M. domestica* and *H. illucens*, with the highest ash content found here, may show potential in combatting mineral and micronutrient deficiency, which are also prevalent and dangerous (30, 31). Also, phosphorus is a mineral sometimes added to aquaculture feed (82), potentially causing water pollution (83). The high mineral content of *M. domestica* emphasizes its suitability as an aquaculture feedstock as well as for human consumption, potentially alleviating phosphorus pollution. The considerably higher levels of neutral and acid detergent fiber identified in *C. butyrospermi* and *M. domestica* compared to *H. illucens* further their consumption potential. The neutral and acid detergent fiber portions of edible insects are thought to contain chitin (84, 85), a molecule which can act as an anticoagulant and can promote wound healing (86).

*Musca domestica* and especially *C. butyrospermi* were also found to possess fatty acid profiles perhaps more suited for human consumption than the other three species. *Cirina butyrospermi* contained by far the highest proportion of poly-unsaturated fatty acids, particularly omega-3 fatty acids, with the original profile produced here being especially rich in α-linolenic acid (ALA). Poly-unsaturated fatty acids have been shown to lower low-density lipoprotein cholesterol levels in men and women (87), protecting against atherosclerotic issues (26) and are also thought essential in child development (24). Dietary deficiencies of poly-unsaturated fatty acids can cause a range of symptoms, including growth inhibition and reduced efficacy of wound healing (88). Conversely, saturated fatty acids, which were highest in *H. illucens*, the species which formed its own fatty acid profile cluster characterized by high saturation, are a risk factor of increased blood pressure, low-density lipoprotein levels, and atherosclerosis (26, 27, 88, 89). Indeed, a food's ratio of poly-unsaturated to saturated fatty acids is often used to indicate its ability to lower cholesterol and reduce the risk of cardiovascular diseases (27). Poly-unsaturated fatty acids are also essential for aquaculture production (25, 29, 90), suggesting that a *C. butyrospermi*-based fish feed may provide the necessary fatty acid profile without the need for dietary supplementation. Meanwhile, *M. domestica* contained the joint highest proportion of mono-unsaturated fatty acids, alongside *R. phoenicis* and *M. bellicosus*, with palmitoleic, elaidic, and oleic acid particularly represented. Mono-unsaturated fatty acids have also been shown to have cholesterol lowering abilities (27, 87) and oleic acid even has anti-carcinogenic properties, suppressing the expression of breast cancer oncogene, *Her-2/neu* (91). Therefore, a diet rich in the shea tree caterpillar, *C. butyrospermi*, and house fly, *M. domestica*, would likely provide a high content of unsaturated fatty acids, bringing multiple associated health benefits. However, an issue with such a diet is that both of these species were found to have the lowest crude fat content whilst the highest was found in *R. phoenicis*.

Fat is the most energy dense nutrient source, producing 9 kcal per gram (92), meaning that low-fat diets may result in calorie deficiency, a leading issue in malnutrition (26, 27). Fat deficiency can also exacerbate protein deficiency due to protein being redirected toward energy metabolism in low fat conditions (26). Furthermore, fats are vital in the structural and biological functioning of cells (60), as well as increasing food palatability through retaining flavors (26, 27, 60). Therefore, supplementation of a *C. butyrospermi*- and *M. domestica*-based diet with high-fat *R. phoenicis* may be ideal. *Rhynchophorus phoenicis* are also seen as a delicacy in many areas and, through breaking down waste in palm agro-systems, can contribute to circularizing palm agronomies in Africa (93), aiding sustainable development.

Furthermore, although not the case for *M. domestica, C. butyrospermi* was found here to be marginally limited in its leucine and methionine content. These essential amino acids are important in energy production by facilitating carnitine synthesis for the transport of fatty acids into the mitochondria for β-oxidation (94); leucine also enhances growth in infants (26). However, the low leucine and methionine in *C. butyrospermi* may be inconsequential because here amino acids are reported relative to crude protein content which was high in this species. Therefore, levels of essential amino acids per unit of *C. butyrospermi*-based food may be expected to more than meet human requirements. All insect species were also shown to contain sufficient lysine, an essential amino acid particularly lacking in staple cereal-based diets (26), which has been shown to have antiviral properties (95).

Therefore, overall these results, although in certain cases with limited sample sizes, suggest that consumption of *C. butyrospermi* and *M. domestica*, with supplementation by the high-fat *R. phoenicis*, may optimize the health benefits of entomophagy. This further supports redressing the current neglect of *C. butyrospermi* in the nutritional literature and promoting investigation of its farming potential as co-production with a growing, and high value, shea-related industry.

### Intraspecific Nutritional Variation

In addition to the between-species nutritional variation discussed above, we also identified substantial within-species variation. For example, the crude protein content of *R. phoenicis* ranged from 10.5 to 66.3% of dry matter and their crude fat from 17.3 to 71.6%, with this fat distribution segregating into two distinct clusters. There are several reasons why such within-species variation may arise.

A first source of variation may stem from differences in the experimental procedures used by different studies as some studies may use non-standard methodology or a differing number of repeats. Here, only studies which nutritionally profiled using standard AOAC (67) methods were incorporated into the dataset in an attempt to reduce experimental variation. However, even within standard procedures there is disagreement in the best way to nutritionally profile edible insects, perhaps due to entomophagy research being relatively novel. For example, many methods for assessing sample crude protein content, including the Kjeldahl method used here, rely on converting nitrogen content to crude protein with a conversion factor (96). A conversion factor of 6.25 has popularly and traditionally been used, stemming from the idea that protein is made up of 16% nitrogen (97). However, this has faced much scrutiny, especially regarding edible insect profiling. This is firstly due to the nitrogen content of protein depending strongly on the amino acid composition (97). Furthermore, the presence of non-protein nitrogen in insects, including in chitin, can cause overestimation of insect crude protein levels (96, 98). Therefore, many have suggested that such conversion factors must be re-evaluated in insect food science. Janssen and Lambertus (96), through comparing the total nitrogen and protein nitrogen of three edible insect species, suggested a new conversion factor of 4.76 for insects. However, Finke (85) argues the opposite, that chitin accounts for a relatively small portion of an insect's nitrogen and is therefore not impacting calculated crude protein values. Indeed, some researchers still favor the conversion factor of 6.25 (29, 99–103) whilst others use 4.76 (104–106). This discrepancy can introduce high protein variation in the literature; for example, Basto, Matos, and Valente (107) calculated *H. illucens* crude protein content with both a conversion factor of 4.76 and 6.25 and obtain values of 32.2 and 46.1%, respectively. Therefore, a consensus must be reached to increase the comparability of the nutritional literature. However, what is there to suggest that a single consensus conversion factor would be appropriate for insects, the most speciose animal group? A different strategy may be for researchers to calculate sample chitin content alongside total nitrogen and scale the nitrogen value accordingly (98). Alternatively, researchers could calculate protein content from amino acid analyses, although this suffers from some methods destroying certain amino acids, such as tryptophan and cysteine being destroyed during acid hydrolysis (108). Regardless, this discussion of contentious conversion factors emphasizes the need to resolve experimental variation so that nutritional consensus can emerge.

As well as between-study analytical variation, the husbandry and rearing conditions or whether insects were farmed or wild-caught, could inflate nutritional variation. Larval diets can greatly affect the insect's nutritional profile, meaning that different substrates in the studies used here could cause marked within-species nutritional divergence. Nutritional divergence between *H. illucens* individuals raised on different diets has been widely reported (63–65). This has implications for the use of edible insects as waste valorisers as well as a source of nutrients as the type of organic waste substrate used can impact the utility of the resultant insects. For example, Scala et al. (109) showed that *H. illucens* raised on apple waste accumulated 50% more fat than those on a mixed diet of fruit and grain waste. Therefore, organic waste of high enough quality may be required for edible insect nutrient profiles suitable for consumption. If heavy supplementation of waste substrates is required, such as with fish offal (65), then the sustainability benefits of circularizing food production systems by raising insects on organic waste side streams may be reduced. Furthermore, differences in the age of harvesting may be inflating the nutritional variation we found. Although only specific life stages were analyzed here, within-life stage nutritional variation has been documented and could be influential. For example, Do et al. (110) showed crude protein differences of around 5% between *H. illucens* larvae with a 3 day age gap, which increased to over 10% with a seven day age gap. Insect farmers and harvesters can thus plan optimal harvesting age for desired nutrient profiles. Finally, rearing conditions may have a large impact on nutrient accumulation, further inflating nutritional variation. Temperature increases can reduce livestock feed intake and conversion efficiency (111, 112), with marked impacts on resultant nutrient profiles. For instance, Dobermann, Field, and Michaelson (113) showed reduced protein content in cricket meal under heat stress. Therefore, with much husbandry variation potentially impacting nutrient profiles, perhaps it is not surprising that any definitive consensus on interspecific nutrient differences is yet to emerge. Quantifying these effects should be a major goal of research going forwards.

Finally, a large contributor to this high within-species variation could be natural or biological variation in individual or population nutrient content. The papers used here isolated insects from a wide range of localities; for example, the original profiles in this paper were produced from individuals from Ghana, whereas Akullo et al. (114) isolated *M. bellicosus* from Uganda. Therefore, nutritional results may differ depending on the specific population being sampled, perhaps due to ancestry or genetic differences. For example, in this study, the *R. phoenicis* crude fat distribution was found to split into two discrete clusters, a high- and low-fat group, potentially reflecting the sampling of two distinct populations or meta-populations. Stark genetic differences have indeed been found between edible insect populations globally. For example, high mitochondrial and microsatellite diversity and structure has been identified in *H. illucens* (115, 116) and *M. domestica* (117, 118). This genetic divergence could potentially translate into nutritional differences. Therefore, perhaps future research, rather than focusing on specific localities, could investigate the geographic and population structuring of nutrient content in order to assess the extent of this biological variation. Beyond that, research could start to use genetic and nutritional variation to allow selective breeding for nutrient content in order to further domesticate and optimize mini-livestock for combatting undernutrition. This has long been practiced in more traditional agro-species. For example, work on cows has identified genetic polymorphisms associated with altered fatty acid profiles, with the potential to select loci in order to reduce saturation (119, 120). There is even interest in the genetic-assisted selective breeding for reduced methane emissions in cows, aiding the cattle industry's sustainability (121). Similar interest in selective breeding has been seen more recently in aquatic agro-species (122, 123). Therefore, research should aim to implement similar methods in edible insect species. Depending on the extent of genotypic and phenotypic variation, the edible insect industry may not be restricted to nutritional levels found naturally, identified in this and other studies. Instead, selective breeding of optimal profiles may be possible, particularly if industrial interest in the commercial rearing of edible insects continues. Many edible insects may be especially suitable from a high-throughput, selective breeding standpoint due to them being manipulable, mass rearable, and with short generation times. However, in order to use genetic techniques for improving nutrient profiles, such as marker-assisted selection (124), the available genetic resources for edible insect species must be increased. For example, although genomes are available for *H. illucens* (125) and *M. domestica* (126), they have not yet been produced for the other three species analyzed here. Therefore, research could do well to make increasing available genetic and nutritional resources a priority.

### Conclusions and Recommendations

Insects can clearly play an important role in future sustainable development and food security, and it is also becoming increasingly clear that this is particularly the case for certain species. Research should continue to characterize the nutrient profiles of as many tractable species as possible to direct interest into promising species in an informed way. For example, here we showed that the shea tree caterpillar, *C. butyrospermi*, alongside the house fly, *M. domestica*, appears particularly nutritionally suitable for human consumption. This is surprising as it is a species that has received little to no research interest. Therefore, continuing to profile this species, including identifying any safety concerns surrounding its consumption, may substantiate the promising results presented here. This is not to say that the other three species analyzed here should not be considered a useful aspect of sustainable food security. As discussed, they may be particularly useful in dietary supplementation, such as the high fat content of *R. phoenicis*. Furthermore, *H. illucens* and *R. phoenicis* show promise in circularizing agro-processes, with *H. illucens* especially popular in the animal feed industry. Furthermore, research could continue to assess the factors dictating individual nutrient content, including husbandry effects such as diet, rearing conditions, sex, and stage of the insect. This would allow insect farmers to optimize nutrient accumulation through husbandry modifications. It may also mean that insect farming moves away from selecting the most nutritious species and toward modifying and optimizing the nutrient content of species which are easily reared or more beneficial for sustainable development. Finally, linked to that is the idea that research could increasingly resolve the extent to which there is natural within-species nutritional variation, as well as any genetic variation underlying this. This could allow the targeted selective breeding of edible insects to create “founder stocks,” which optimize their nutrient profiles for combatting undernutrition and assuring sustainable food security.

## Data Availability Statement

The original contributions presented in the study are included in the article/Supplementary Material, further inquiries can be directed to the corresponding author/s.

## Author Contributions

JA designed the research and carried out fieldwork. XC and IO conducted the biochemical analyses. JA and XC analyzed the data. BR conducted the statistical and meta-analyses. JA and BR wrote the initial manuscript, with contributions from VS and CC. All authors contributed to the article and approved the submitted version.

## Funding

This study received funding contributions from the University of Michigan PARTNER II, AnePaare Farms, Aspire Food Group, the Association of African Universities (AAU), Research England's Global Challenges Research Fund (GCRF), and the Royal Society (UK).

## Conflict of Interest

The authors declare that the research was conducted in the absence of any commercial or financial relationships that could be construed as a potential conflict of interest.

## Publisher's Note

All claims expressed in this article are solely those of the authors and do not necessarily represent those of their affiliated organizations, or those of the publisher, the editors and the reviewers. Any product that may be evaluated in this article, or claim that may be made by its manufacturer, is not guaranteed or endorsed by the publisher.
