# Supplementary material for: The Nutritional Profiles of Five Important Edible Insect Species From West Africa—An Analytical and Literature Synthesis
Source: Front Nutr. 2021 Dec 3;8:792941. doi: 10.3389/fnut.2021.792941 (PMC8678595; doi:10.3389/fnut.2021.792941)
Supplement: Supplementary file 1 [file Table_1.DOCX]

Table S1: The literature used in this study’s nutritional meta-analysis, along with the species profiled by each paper and the dataset to which they contributed profiles; either proximate, fatty acid, or amino acid.

| Citation | Species | Proximate | Fatty Acid | Amino Acid |
| --- | --- | --- | --- | --- |
| Abdel-Tawwab et al. (1) | BSF | ✓ |  |  |
| Adeboye et al. (2) | TM | ✓ |  |  |
| Ademola et al. (3) | PL |  |  | ✓ |
| Ademola et al. (3) | TM |  |  | ✓ |
| Adeyeye et al. (4) | TM | ✓ |  |  |
| Akakpo et al. (5) | PL | ✓ |  |  |
| Akakpo et al. (6) | TM | ✓ |  |  |
| Akullo et al. (7) | TM | ✓ | ✓ |  |
| Anvo et al. (8) | STC | ✓ | ✓ | ✓ |
| Awobusuyi et al. (9) | TM | ✓ |  |  |
| Banjo et al. (10) | PL | ✓ |  |  |
| Banjo et al. (10) | TM | ✓ |  |  |
| Barroso et al. (11) | BSF | ✓ | ✓ |  |
| Barroso et al. (11) | HSF | ✓ | ✓ |  |
| Barroso et al. (12) | BSF | ✓ | ✓ |  |
| Barroso et al. (13) | BSF | ✓ | ✓ |  |
| Basto et al. (14) | BSF | ✓ |  |  |
| Bejaei et al. (15) | BSF | ✓ |  |  |
| Belghit et al. (16) | BSF |  |  | ✓ |
| Bosch et al. (17) | BSF | ✓ |  | ✓ |
| Bosch et al. (17) | HSF |  |  | ✓ |
| Bussler, Rumpold et al. (18) | BSF | ✓ |  |  |
| Campbell et al. (19) | BSF | ✓ |  |  |
| Chia et al. (20) | BSF | ✓ |  |  |
| Chia et al. (21) | BSF | ✓ |  |  |
| Chu et al. (22) | BSF | ✓ |  |  |
| De Marco et al. (23) | BSF | ✓ |  |  |
| Dzepe et al. (24) | BSF | ✓ |  |  |
| Dzepe et al. (24) | HSF | ✓ |  |  |
| Edijala et al. (25) | PL | ✓ |  |  |
| Ekop et al. (26) | PL | ✓ |  |  |
| Ekpo et al. (27) | PL |  | ✓ |  |
| Ekpo et al. (27) | TM |  | ✓ |  |
| Ekpo, K. (28) | PL | ✓ |  | ✓ |
| Ekpo, K. (28) | TM | ✓ |  | ✓ |
| Elemo et al. (29) | PL | ✓ | ✓ |  |
| Fitches et al. (30) | HSF | ✓ |  |  |
| Gana et al. (31) | BSF | ✓ |  |  |
| Giannetto et al. (32) | BSF | ✓ |  |  |
| Gold et al. (33) | BSF | ✓ |  |  |
| Gonzalez et al. (34) | BSF | ✓ |  |  |
| Hussein et al. (35) | HSF | ✓ | ✓ | ✓ |
| Idolo (36) | PL | ✓ |  |  |
| Idowu et al. (37) | TM | ✓ |  |  |
| Igwe et al. (38) | PL | ✓ |  | ✓ |
| Jankowski et al. (39) | BSF | ✓ |  |  |
| Jayanegara et al. (40) | BSF |  | ✓ |  |
| Jonathan (41) | PL | ✓ |  |  |
| Jucker et al. (42) | BSF | ✓ |  |  |
| Kim et al. (43) | BSF | ✓ |  |  |
| Kim et al. (44) | BSF |  | ✓ |  |
| Kim et al. (45) | BSF |  | ✓ |  |
| Kinyuru et al. (46) | TM | ✓ | ✓ |  |
| Koffi et al. (47) | PL | ✓ |  |  |
| Kovitvadhi et al. (48) | HSF | ✓ |  |  |
| Kovitvadhi et al. (49) | HSF | ✓ |  |  |
| Larouche et al. (50) | BSF | ✓ |  |  |
| Lawal et al. (51) | BSF | ✓ | ✓ |  |
| Leni et al. (52) | BSF | ✓ |  | ✓ |
| Liland et al. (53) | BSF | ✓ | ✓ | ✓ |
| Liu et al. (54) | BSF | ✓ | ✓ |  |
| Liu et al. (55) | BSF | ✓ |  |  |
| MarkManuel et al. (56) | PL | ✓ |  |  |
| Mastoraki et al. (57) | HSF | ✓ |  |  |
| Matthaus et al. (58) | BSF | ✓ |  |  |
| Mba et al. (59) | PL | ✓ | ✓ | ✓ |
| McCusker et al. (60) | BSF | ✓ |  |  |
| Mintah et al. (61) | BSF | ✓ |  |  |
| Nzikou et al. (62) | PL | ✓ | ✓ |  |
| Ogbuagu et al. (63) | PL |  | ✓ | ✓ |
| Okaraonye et al. (64) | PL | ✓ | ✓ | ✓ |
| Okunowu et al. (65) | PL | ✓ | ✓ | ✓ |
| Olaleye (66) | TM |  |  | ✓ |
| Omotoso et al. (67) | PL | ✓ |  |  |
| Onyeike et al. (68) | PL | ✓ |  |  |
| Opara et al. (69) | PL | ✓ |  |  |
| Paiko et al. (70) | TM | ✓ | ✓ | ✓ |
| Pieterse et al. (71) | HSF | ✓ | ✓ |  |
| Qi et al. (72) | HSF | ✓ |  |  |
| Quaye et al. (73) | PL | ✓ |  |  |
| Rabani et al. (74) | BSF |  | ✓ |  |
| Rahul et al. (75) | BSF | ✓ |  | ✓ |
| Ramos-Bueno et al. (76) | BSF |  | ✓ |  |
| Reyes et al. (77) | BSF |  | ✓ |  |
| Riudavets et al. (78) | BSF | ✓ |  |  |
| Roberts et al. (79) | BSF | ✓ |  |  |
| Saleh (80) | HSF | ✓ |  |  |
| Sallau et al. (81) | TM | ✓ |  |  |
| Shindi et al. (82) | TM | ✓ |  |  |
| Shumo et al. (83) | BSF | ✓ |  |  |
| Smets et al. (84) | BSF | ✓ | ✓ | ✓ |
| Smets et al. (85) | BSF |  | ✓ |  |
| Song-QuanOng et al. (86) | HSF | ✓ |  |  |
| Tippayadara et al. (87) | BSF | ✓ |  |  |
| Traksele et al. (88) | BSF | ✓ |  |  |
| Wallace et al. (89) | BSF | ✓ |  |  |
| Wang et al. (90) | BSF |  | ✓ |  |
| Womeni et al. (91) | PL | ✓ | ✓ |  |
| Womeni et al. (92) | PL | ✓ |  | ✓ |
| Wong et al. (93) | BSF | ✓ |  |  |
| Yang et al. (94) | HSF | ✓ | ✓ |  |
| Yapo et al. (95) | STC | ✓ | ✓ | ✓ |
| Zi-zhe et al. (96) | HSF |  | ✓ |  |
| *BSF=black soldier fly; HSF=house fly; PL=African palm weevil; STC=shea tree caterpillar; TM=African termite* | | | | |

References:

1. Abdel-Tawwab M, Khalil RH, Metwally AA, Shakweer MS, Khallaf MA, Abdel-Latif HMR. Effects of black soldier fly (Hermetia illucens L.) larvae meal on growth performance, organs-somatic indices, body composition, and hemato-biochemical variables of European sea bass, Dicentrarchus labrax. (2020) doi:10.1016/j.aquaculture.2020.735136

2. Adeboye AO, Fasogbon BM, Adegbuyi K. Formulation of vegetable soup powder from Clerodendrum volubile enriched with Macrotermes bellicosus (termite) flour. *Int J Trop Insect Sci* (2020) doi:10.1007/s42690-020-00350-1

3. Ademola OA, Omolara AH, Abioye OR. Amino Acids Profile of Bee Brood, Soldier Termite, Snout Beetle Larva, Silkworm Larva and Pupa: Nutritional Implications. *Adv Anal Chem* (2017) **7**:31–38. doi:10.5923/j.aac.20170702.02

4. Adeyeye EI, Olaleye AA. Chemical composition and mineral safety index of five insects commonly eaten in South West Nigeria. *FUW Trends Sci Technol J* (2016) **1**:139–144.

5. Akakpo AY, Tchaniley L, Osseyi EG, Tchacondo T. Assessment of the nutritional value and the quality of the oil of Macrotermes bellicosus insect collected during Rain season in Togo. *Int J Entomol Res* (2020) **5**:104–109.

6. Akakpo AY, Tchaniley L, Osseyi EG, Tchacondo T. Biochemical composition and nutritional value of larvae of Rhynchophorus phoenicis collected from rot palm trees (Elaeis guineensis) in palm groves in Togo. *Am J Innov Res Appl Sci* (2020)

7. Akullo, Agea, Obaa, Okwee-Acai, Nakimbugwe D. Nutrient composition of commonly consumed edible insects in the Lango sub-region of northern Uganda. *Int Food Res J* (2018) **25**:159–165.

8. Anvo PM, Toguyéni A, Otchoumou AK, Zoungrana-Kaboré CY, Koumelan EP. Nutritional qualities of edible caterpillars Cirina butyrospermi in southwestern of Burkina Faso. *Int J Innov Appl Stud* (2016) **18**:639–645. Available at: http://www.ijias.issr-journals.org/abstract.php?article=IJIAS-16-200-17

9. Awobusuyi TD, Siwela M, Pillay K. Nutritional composition of insect types most commonly consumed by the Olugboja Community of Ondo State, Nigeria. *Int J Trop Insect Sci* (2021) doi:10.1007/s42690-021-00483-x

10. Banjo AD, Lawal OA, Songonuga EA. The nutritional value of fourteen species of edible insects in southwestern Nigeria. *African J Biotechnol* (2006) **5**:298–301. Available at: http://www.academicjournals.org/AJB

11. Barroso FG, de Haro C, Sánchez-Muros MJ, Venegas E, Martínez-Sánchez A, Pérez-Bañón C. The potential of various insect species for use as food for fish. *Aquaculture* (2014) **422**–**423**:193–201. doi:10.1016/j.aquaculture.2013.12.024

12. Barroso FG, Sánchez-Muros M-J, Segura M, Morote E, Torres A, Ramos R, Guil J-L. Insects as food: Enrichment of larvae of Hermetia illucens with omega 3 fatty acids by means of dietary modifications. *J Food Compos Anal* (2017) **62**:8–13. doi:10.1016/j.jfca.2017.04.008

13. Barroso FG, Sánchez-Muros MJ, Rincón MÁ, Rodriguez-Rodriguez M, Fabrikov D, Morote E, Guil-Guerrero JL. Production of n-3-rich insects by bioaccumulation of fishery waste. *J Food Compos Anal* (2019) **82**:103237. doi:10.1016/j.jfca.2019.103237

14. Basto A, Matos E, Valente LMP. Nutritional value of different insect larvae meals as protein sources for European sea bass (Dicentrarchus labrax) juveniles. *Aquaculture* (2020) **521**:735085. doi:10.1016/j.aquaculture.2020.735085

15. Bejaei M, Cheng KM. The effect of including full-fat dried black soldier fly larvae in laying hen diet on egg quality and sensory characteristics. *J Insects as Food Feed* (2020) **6**:305–314. doi:10.3920/JIFF2019.0045

16. Belghit I, Lock E-J, Fumière O, Lecrenier M-C, Renard P, Dieu M, Berntssen MHG, Palmblad M, Rasinger JD. Species-Specific Discrimination of Insect Meals for Aquafeeds by Direct Comparison of Tandem Mass Spectra. *Animals* (2019) **9**:222. doi:10.3390/ani9050222

17. Bosch G, Zhang S, Oonincx DGAB, Hendriks WH. Protein quality of insects as potential ingredients for dog and cat foods. *J Nutr Sci* (2014) **3**:e29. doi:10.1017/jns.2014.23

18. Bußler S, Rumpold BA, Jander E, Rawel HM, Schlüter OK. Recovery and techno-functionality of flours and proteins from two edible insect species: Meal worm (Tenebrio molitor) and black soldier fly (Hermetia illucens) larvae. *Heliyon* (2016) **2**: doi:10.1016/j.heliyon.2016.e00218

19. Campbell M, Ortuño J, Stratakos AC, Linton M, Corcionivoschi N, Elliott T, Koidis A, Theodoridou K. Impact of Thermal and High-Pressure Treatments on the Microbiological Quality and In Vitro Digestibility of Black Soldier Fly (Hermetia illucens) Larvae. *Animals* (2020) **10**:682. doi:10.3390/ani10040682

20. Chia SY, Tanga CM, Osuga IM, Alaru AO, Mwangi DM, Githinji M, Subramanian S, Fiaboe KKM, Ekesi S, van Loon JJA, et al. Effect of Dietary Replacement of Fishmeal by Insect Meal on Growth Performance, Blood Profiles and Economics of Growing Pigs in Kenya. *Animals* (2019) **9**:705. doi:10.3390/ani9100705

21. Chia SY, Tanga CM, Osuga IM, Cheseto X, Ekesi S, Dicke M, van Loon JJA. Nutritional composition of black soldier fly larvae feeding on agro-industrial by-products. *Entomol Exp Appl* (2020) **168**:472–481. doi:10.1111/eea.12940

22. Chu X, Li M, Wang G, Wang K, Shang R, Wang Z, Li L. Evaluation of the Low Inclusion of Full-Fatted Hermetia illucens Larvae Meal for Layer Chickens: Growth Performance, Nutrient Digestibility, and Gut Health. *Front Vet Sci* (2020) **7**:585843. doi:10.3389/fvets.2020.585843

23. De Marco M, Martínez S, Hernandez F, Madrid J, Gai F, Rotolo L, Belforti M, Bergero D, Katz H, Dabbou S, et al. Nutritional value of two insect larval meals (Tenebrio molitor and Hermetia illucens) for broiler chickens: Apparent nutrient digestibility, apparent ileal amino acid digestibility and apparent metabolizable energy. *Anim Feed Sci Technol* (2015) **209**:211–218. doi:10.1016/j.anifeedsci.2015.08.006

24. Dzepe D, Magatsing O, Kuietche HM, Meutchieye F, Nana P, Tchuinkam T, Djouaka R. Recycling Organic Wastes Using Black Soldier Fly and House Fly Larvae as Broiler Feed. *Circ Econ Sustain* (2021) doi:10.1007/s43615-021-00038-9

25. Edijala JK, Egbogbo O, Anigboro AA. Proximate composition and cholesterol concentrations of Rhynchophorus phoenicis and Oryctes monoceros larvae subjected to different heat treatments. *African J Biotechnol* (2009) **8**:2346–2348. doi:10.4314/ajb.v8i10.60595

26. Ekop EA, Udoh AI, Akpan PE. Proximate and anti-nutrient composition of four edible insects in Akwa Ibom State, Nigeria. *World J Appl Sci Technol* (2010) **2**:224–231. Available at: www.wojast.com

27. Ekpo KE, Onigbinde AO, Asia IO. Pharmaceutical potentials of the oils of some popular insects consumed in southern Nigeria. *African J Pharm Pharmacol* (2009) **3**:51–57.

28. Ekpo KE. Effect of processing on the protein quality of four popular insects consumed in Southern Nigeria. *Arch Appl Sci Res* (2011) **3**:307–326.

29. Elemo BO, Elemo GN, Makinde MA, Erukainure OL. Chemical evaluation of African palm weevil, Rhychophorus phoenicis, larvae as a food source. *J Insect Sci* (2011) **11**:1–6. doi:10.1673/031.011.14601

30. Fitches EC, Dickinson M, De Marzo D, Wakefield ME, Charlton AC, Hall H. Alternative protein production for animal feed: Musca domestica productivity on poultry litter and nutritional quality of processed larval meals. *J Insects as Food Feed* (2018) **5**:77–88. doi:10.3920/JIFF2017.0061

31. Gana AB, Tijani S, Ibrahim R. Nutrient utilization , haematological indices and carcass compositions of heteroclarias fingerlings fed Hermetia illucens (Linnaeus , 1758) larvae meal. *Int J Fish Aquat Stud* (2020) **8**:42–48.

32. Giannetto A, Oliva S, Lanes CFC, de Araújo Pedron F, Savastano D, Baviera C, Parrino V, Lo Paro G, Spanò NC, Cappello T, et al. Hermetia illucens (Diptera: Stratiomydae) larvae and prepupae: Biomass production, fatty acid profile and expression of key genes involved in lipid metabolism. *J Biotechnol* (2020) **307**:44–54. doi:10.1016/j.jbiotec.2019.10.015

33. Gold M, Cassar CM, Zurbrügg C, Kreuzer M, Boulos S, Diener S, Mathys A. Biowaste treatment with black soldier fly larvae: Increasing performance through the formulation of biowastes based on protein and carbohydrates. *Waste Manag* (2020) **102**:319–329. doi:10.1016/j.wasman.2019.10.036

34. González CM, Garzón R, Rosell CM. Insects as ingredients for bakery goods. A comparison study of H. illucens, A. domestica and T. molitor flours. *Innov Food Sci Emerg Technol* (2019) **51**:205–210. doi:10.1016/j.ifset.2018.03.021

35. Hussein M, Pillai V V., Goddard JM, Park HG, Kothapalli KS, Ross DA, Ketterings QM, Brenna JT, Milstein MB, Marquis H, et al. Sustainable production of housefly (Musca domestica) larvae as a protein-rich feed ingredient by utilizing cattle manure. *PLoS One* (2017) **12**:e0171708. doi:10.1371/journal.pone.0171708

36. Idolo I. Nutritional and quality attributes of wheat buns enriched with the larvae of Rhynchophorus phoenicis f. *Pakistan J Nutr* (2010) **9**:1043–1046. doi:10.3923/pjn.2010.1043.1046

37. Idowu AB, Oliyide EO, Ademolu KO, Bamidele JA. Nutritional and anti-nutritional evaluation of three edible insects consumed by the Abeokuta community in Nigeria. *Int J Trop Insect Sci* (2019) **39**:157–163. doi:10.1007/s42690-019-00021-w

38. Igwe CU. Assessment of the Protein Quality of Some Edible Insects And Mollusks as Potential Food Sources. *FUTO J Ser* (2015) **1**:196–206.

39. Jankowski J, Kozłowski K, Zdunczyk Z, Stępniowska A, Ognik K, Kieronczyk B, Józefiak D, Juskiewicz J. The effect of dietary full-fat Hermetia illucens larvae meal on gut physiology and growth performance in young turkeys. *Anim Feed Sci Technol* (2021) **275**:114879. doi:10.1016/j.anifeedsci.2021.114879

40. Jayanegara A, Gustanti R, Ridwan R, Widyastuti Y. Fatty acid profiles of some insect oils and their effects on in vitro bovine rumen fermentation and methanogenesis. *Ital J Anim Sci* (2020) **19**:1310–1317. doi:10.1080/1828051X.2020.1841571

41. Jonathan AA. Proximate and anti-nutritional composition of two common edible insects: yam beetle (Heteroligus meles) and palm weevil (Rhynchophorus phoenicis). *Elixir Food Sci* (2012) **49**:9782–9786.

42. Jucker C, Lupi D, Moore CD, Leonardi MG, Savoldelli S. Nutrient Recapture from Insect Farm Waste: Bioconversion with Hermetia illucens (L.) (Diptera: Stratiomyidae). *Sustainability* (2020) **12**:362. doi:10.3390/su12010362

43. Kim SW, Jung TS, Ha YJ, Gal SW, Noh CW, Kim IS, Lee JH, Yoo JH. Removal of fat from crushed black soldier fly larvae by carbon dioxide supercritical extraction. *J Anim Feed Sci* (2019) **28**:83–88. doi:10.22358/jafs/105132/2019

44. Kim B, Bang HT, Kim KH, Kim MJ, Jeong JY, Chun JL, Ji SY. Evaluation of black soldier fly larvae oil as a dietary fat source in broiler chicken diets. *J Anim Sci Technol J Anim Sci Technol* (2020) **62**:187–197. doi:10.5187/jast.2020.62.2.187

45. Kim YB, Kim DH, Jeong SB, Lee JW, Kim TH, Lee HG, Lee KW. Black soldier fly larvae oil as an alternative fat source in broiler nutrition. *Poult Sci* (2020) **99**:3133–3143. doi:10.1016/j.psj.2020.01.018

46. Kinyuru JN, Konyole SO, Roos N, Onyango CA, Owino VO, Owuor BO, Estambale BB, Friis H, Aagaard-Hansen J, Kenji GM. Nutrient composition of four species of winged termites consumed in western Kenya. *J Food Compos Anal* (2013) **30**:120–124. doi:10.1016/j.jfca.2013.02.008

47. Koffi DM, Cisse M, Koua GA, Niamke SL. Nutritional and functional properties of flour from the palm (Elaeis Guineensis) weevil Rhynchophorus phoenicis larvae consumed as protein source in South Côte D’Ivoire. *Ann Univ Dunarea Jos Galati, Fascicle VI Food Technol* (2017) **41**:9–19.

48. Kovitvadhi A, Chundang P, Thongprajukaew K, Tirawattanawanich C, Srikachar S, Chotimanothum B. Potential of Insect Meals as Protein Sources for Meat-Type Ducks Based on In Vitro Digestibility. *Animals* (2019) **9**:155. doi:10.3390/ani9040155

49. Kovitvadhi A, Chundang P, Pliantiangtam N, Thongprajukaew K, Tirwattanawanich C, Suwanasopee T, Koonawootrittriron S. Screening of in vitro nutrient digestibility coefficients of selected insect meals in broiler chickens, black-meat chickens and quails. *Anim Physiol Anim Nutr* (2021) **105**:305–315. doi:10.1111/jpn.13451

50. Larouche J, Deschamps MH, Saucier L, Lebeuf Y, Doyen A, Vandenberg GW. Effects of Killing Methods on Lipid Oxidation, Colour and Microbial Load of Black Soldier Fly (Hermetia illucens) Larvae. *Animals* (2019) **9**:182. doi:10.3390/ani9040182

51. Lawal KG, Kavle RR, Akanbi TO, Mirosa M, Agyei D. Enrichment in specific fatty acids profile of Tenebrio molitor and Hermetia illucens larvae through feeding. *Futur Foods* (2021) **3**:100016. doi:10.1016/j.fufo.2021.100016

52. Leni G, Soetemans L, Jacobs J, Depraetere S, Ginaotten N, Bastiaens L, Caligiani A, Sforza S. Protein hydrolysates from Alphitobius diaperinus and Hermetia illucens larvae treated with commercial proteases. *J Insects as Food Feed* (2020) **6**:393–404. doi:10.3920/JIFF2019.0037

53. Liland NS, Biancarosa I, Araujo P, Biemans D, Bruckner CG, Waagbø R, Torstensen BE, Lock EJ. Modulation of nutrient composition of black soldier fly (Hermetia illucens) larvae by feeding seaweed-enriched media. *PLoS One* (2017) **12**:e0183188. doi:10.1371/journal.pone.0183188

54. Liu X, Chen X, Wang H, Yang Q, ur Rehman K, Li W, Cai M, Li Q, Mazza L, Zhang J, et al. Dynamic changes of nutrient composition throughout the entire life cycle of black soldier fly. *PLoS One* (2017) **12**:e0182601. doi:10.1371/journal.pone.0182601

55. Liu X, Liu X, Yao Y, Qu X, Chen J, Xie K, Wang X, Qi Y, Xiao B, He C, et al. Effects of different levels of Hermetia illucens larvae meal on performance, egg quality, yolk fatty acid composition and oxidative status of laying hens. *Ital J Anim Sci* (2021) **20**:256–266. doi:10.1080/1828051X.2021.1878946

56. MarkManuel DP, Godwin J. Effects of Culinary Methods on The Proximate Composition of an Edible Insect (Rhynchophorus Phoenicis) Larvae Obtained From Bayelsa State, Nigeria. *Eur J Agric Food Sci* (2020) **2**:1–7. doi:10.24018/ejfood.2020.2.4.67

57. Mastoraki M, Vlahos N, Patsea E, Chatzifotis S, Mente E, Antonopoulou E. The effect of insect meal as a feed ingredient on survival, growth, and metabolic and antioxidant response of juvenile prawn Palaemon adspersus (Rathke, 1837). *Aquac Res* (2020) **51**:3551–3562. doi:10.1111/are.14692

58. Matthäus B, Piofczyk T, Katz H, Pudel F. Renewable Resources from Insects: Exploitation, Properties, and Refining of Fat Obtained by Cold-Pressing from Hermetia illucens (Black Soldier Fly) Larvae. *Eur J Lipid Sci Technol* (2018) **121**:1800376. doi:10.1002/ejlt.201800376

59. Mba ARF, Kansci G, Viau M, Hafnaoui N, Meynier A, Demmano G, Genot C. Lipid and amino acid profiles support the potential of Rhynchophorus phoenicis larvae for human nutrition. *J Food Compos Anal* (2017) **60**:64–73. doi:10.1016/j.jfca.2017.03.016

60. Mccusker S, Buff PR, Yu Z, Fascetti AJ. Amino acid content of selected plant, algae and insect species: a search for alternative protein sources for use in pet foods. *J Nutr Sci* (2014) **3**:1–5. doi:10.1017/jns.2014.33

61. Mintah BK, He R, Agyekum AA, Dabbour M, Golly MK, Ma H. Edible insect protein for food applications: Extraction, composition, and functional properties. *J Food Process Eng* (2020) **43**: doi:10.1111/jfpe.13362

62. Nzikou JM, Mbemba F, Mvoula-Tsieri M, Diabangouaya-Batéla B, Malela KE, Kimbonguila A, Ndangui CB, Pambou-Tobi NP, Silou T, Desobry S. Characterisation and Nutritional Potentials of Rhynchophorus phoenicis Larva Consumed in Congo-Brazzaville. *Curr Res J Biol Sci* (2010) **2**:189–194.

63. Ogbuagu MN, Ohondu I, Nwigwe C. Fatty Acid and Amino Acid Profiles of the Larva of Raffia Palm Weevil: Rhynchophorus phoenicis. (2011). Available at: http://www.akamaiuniversity.us/PJST.htm [Accessed April 26, 2021]

64. Okaraonye CC, Ikewuchi JC. Rhynchophorus phoenicis (F) larva meal: Nutritional value and health implications. *J Biol Sci* (2008) **8**:1221–1225. doi:10.3923/jbs.2008.1221.1225

65. Okunowo WO, Olagboye AM, Afolabi LO, Oyedeji AO. Nutritional Value of Rhynchophorus phoenicis (F.) Larvae, an Edible Insect in Nigeria. *African Entomol* (2017) **25**:156–163. doi:10.4001/003.025.0156

66. Olaleye AA. Amino acid profiles of five commonly consumed insects in southwestern Nigeria. *Carpathian J Food Sci Technol* (2020) **12**:42–51. doi:10.34302/crpjfst/2020.12.5.3

67. Omotoso OT, Adedire CO. Nutrient composition, mineral content and the solubility of the proteins of palm weevil, Rhynchophorus phoenicis f. (Coleoptera: Curculionidae). *J Zhejiang Univ Sci B* (2007) **8**:318–322. doi:10.1631/jzus.2007.B0318

68. Onyeike EN, Ayalogu EO, Okaraonye CC. Nutritive value of the larvae of raphia palm beetle (Oryctes rhinoceros) and weevil (Rhyncophorus pheonicis). *J Sci Food Agric* (2005) **85**:1822–1828. doi:10.1002/jsfa.2054

69. Opara MN, Sanyigha FT, Okoli. Studies on the production trend and quality characteristics of palm grubs in the tropical rainforest zone of Nigeria. *J Agric Technol* (2012) **8**:851–860. Available at: http://www.ijat-aatsea.com

70. Paiko YB, Azeh Y, Ibrahim IL, Awwal IL. Nutritional composition of Edible Termites (Macrotermis bellicosus) Consumed in Paikoro Local Government, Niger State, Nigeria. *Lapai J Appl Nat Sci* (2018) **3**:214–221.

71. Pieterse E, Pretorius Q. Nutritional evaluation of dried larvae and pupae meal of the housefly (Musca domestica) using chemical-and broiler-based biological assays. *Anim Prod Sci* (2014) **54**:347–355. doi:10.1071/AN12370

72. Qi X, Li Z, Akami M, Mansour A, Niu C. Fermented crop straws by Trichoderma viride and Saccharomyces cerevisiae enhanced the bioconversion rate of Musca domestica (Diptera: Muscidae). *Environ Sci Pollut Res* (2019) **26**:29388–29396. doi:10.1007/s11356-019-06101-1

73. Quaye B, Atuahene CC, Donkoh A, Adjei BM, Opoku O, Amankrah MA. Nutritional Potential and Microbial Status of African Palm Weevil ( Rhynchophorus phoenicis ) Larvae Raised on Alternative Feed Resources. *Am Sci Res J Eng Technol Sci* (2018) **48**:45–52.

74. Rabani V, Cheatsazan H, Davani S. Proteomics and Lipidomics of Black Soldier Fly (Diptera: Stratiomyidae) and Blow Fly (Diptera: Calliphoridae) Larvae. *J Insect Sci* (2019) **19**:1–9. doi:10.1093/jisesa/iez050

75. Rahul A, Kumar MK, Selvam SP. Edible Insects As An Alternate Source of Complete Nutrition. *Int J Biotech Trends Technol* (2020) **10**:1–5. doi:10.14445/22490183/ijbtt-v10i4p601

76. Ramos-Bueno RP, González-Fernández MJ, Sánchez-Muros-Lozano MJ, García-Barroso F, Guil-Guerrero JL. Fatty acid profiles and cholesterol content of seven insect species assessed by several extraction systems. *Eur Food Res Technol* (2016) **242**:1471–1477. doi:10.1007/s00217-016-2647-7

77. Reyes M, Rodríguez M, Montes J, Barroso FG, Fabrikov D, Morote E, Sánchez-Muros MJ. Nutritional and Growth Effect of Insect Meal Inclusion on Seabass (Dicentrarchuss labrax) Feeds. *Fishes* (2020) **5**: doi:10.3390/fishes5020016

78. Riudavets J, Castañé C, Agustí N, del Arco L, Diaz I, Castellari M. Development and Biomass Composition of Ephestia kuehniella (Lepidoptera: Pyralidae), Tenebrio molitor (Coleoptera: Tenebrionidae), and Hermetia illucens (Diptera: Stratiomyidae) Reared on Different Byproducts of the Agri-Food Industry. *J Insect Sci* (2020) **20**:1–8. doi:10.1093/jisesa/ieaa085

79. Roberts AD, Elly S, Martin L, Michael D. Bioconversion of Fermented Kitchen Waste or Sweet Potato Roots by Black Soldier Fly (Hermetia illucens) Larvae in an Open Shed Environment. (2019) **22**:1–9. Available at: https://www.researchgate.net/publication/339445865

80. Saleh HHE. Effect of Feeding on Fresh (wet) Housefly Maggots (Musca domestica) with or without Artificial Diet on Water Quality and Growth Rates of African Catfish (Clarias gariepinus Burchell, 1822) Fry under Laboratory Conditions. *J Zool Res* (2020) **2**: doi:10.30564/jzr.v2i2.2053

81. Sallau AB, Mada SB, Biola MA. Physicochemical Properties of Oil Extracted from Winged Termite Macrotermis bellicosus. *Int J Mod Biochem* (2012) **1**:36–40. Available at: https://www.researchgate.net/publication/269216485 [Accessed April 15, 2021]

82. Shindi HA, Majeed Q, Bandiya HM, Yahaya MM, Aiki I. Comparing the Nutritional Composition of Some Edible Insects and Some Animal Meats in North-western State of Nigeria. *Asian J Res Zool* (2019) **2**:1–5. doi:10.9734/ajriz/2019/v2i330070

83. Shumo M, Osuga IM, Khamis FM, Tanga CM, Fiaboe KKM, Subramanian S, Ekesi S, van Huis A, Borgemeister C. The nutritive value of black soldier fly larvae reared on common organic waste streams in Kenya. *Sci Rep* (2019) **9**: doi:10.1038/s41598-019-46603-z

84. Smets R, Verbinnen B, Van De Voorde I, Aerts G, Claes J, Van Der Borght M. Sequential Extraction and Characterisation of Lipids, Proteins, and Chitin from Black Soldier Fly (Hermetia illucens) Larvae, Prepupae, and Pupae. *Waste and Biomass Valorization* (2020) **11**:6455–6466. doi:10.1007/s12649-019-00924-2

85. Smets R, Goos P, Claes J, Van Der Borght M. Optimisation of the lipid extraction of fresh black soldier fly larvae (Hermetia illucens) with 2-methyltetrahydrofuran by response surface methodology. *Sep Purif Technol* (2021) **258**:118040. doi:10.1016/j.seppur.2020.118040

86. Song-QuanOng, Ahmad H. Evaluating the biological conversion of paddy husk to house fly, Musca domestica L. (Diptera: Muscidae) pupa powder: a study of house fly as animal feed. *Serangga* (2015) **20**:1–12.

87. Tippayadara N, Dawood MAO, Krutmuang P, Hoseinifar SH, Van Doan H, Paolucci M. Replacement of Fish Meal by Black Soldier Fly (Hermetia illucens) Larvae Meal: Effects on Growth, Haematology, and Skin Mucus Immunity of Nile Tilapia, Oreochromis niloticus. *Animals* (2021) **11**:193. doi:10.3390/ani11010193

88. Traksele L, Speiciene V, Smicius R, Alencikiene G, Salaseviciene A, Garmiene G, Zigmantaite V, Grigaleviciute R. Investigation of in vitro and in vivo digestibility of black soldier fly (Hermetia illucens L.) larvae protein. *J Funct Foods* (2021) **79**:104402.

89. Wallace PA, Nyameasem JK, Aboagye GA, Affedzie-Obresi S, Nkegbe K, Murray F, Botchway V, Karbo N, Leschen W, Maquart P, et al. Effects of replacing fishmeal with black soldier fly larval meal in the diets of grower-finishing guinea fowls reared under tropical conditions. *Trop Anim Health Prod* (2018) **50**:1499–1507. doi:10.1007/s11250-018-1588-5

90. Wang C, Qian L, Wang W, Wang T, Deng Z, Yang F, Xiong J, Feng W. Exploring the potential of lipids from black soldier fly: New paradigm for biodiesel production (I). *Renew Energy* (2017) **111**:749–756. doi:10.1016/j.renene.2017.04.063

91. Womeni HM, Linder M, Tiencheu B, Mbiapo FT, Villeneuve P, Fanni J, Parmentier M. Oils of insects and larvae consumed in Africa: Potential sources of polyunsaturated fatty acids. *OCL - Ol Corps Gras Lipides* (2009) **16**:230–235. doi:10.1684/ocl.2009.0279

92. Womeni HM, Tiencheu B, Linder M, Nabayo EMC, Tenyang N, Mbiapo FT, Villeneuve P, Fanni J, Parmentier M. Nutritional value and effect of cooking, drying and storage process on some functional properties of Rhynchophorus phoenicis. *Int J Life Sci Pharma Res* (2012) **2**:203–219.

93. Wong CY, Rosli SS, Uemura Y, Ho YC, Leejeerajumnean A, Kiatkittipong W, Cheng CK, Lam MK, Lim JW. Potential Protein and Biodiesel Sources from Black Soldier Fly Larvae: Insights of Larval Harvesting Instar and Fermented Feeding Medium. *Energies* (2019) **12**:1570. doi:10.3390/en12081570

94. Yang S, Li Q, Gao Y, Zheng L, Liu Z. Biodiesel production from swine manure via housefly larvae (Musca domestica L.). *Renew Energy* (2014) **66**:222–227. doi:10.1016/j.renene.2013.11.076

95. Yapo M, Amara M, Tuo Y. Nutritional value of shea caterpillar (Cirina butyspermii Vuillet) sold at the market of Korhogo (Côte d’Ivoire). *Int J Agron Agric Res* (2017) **10**:35–44.

96. Zi-zhe C, De-po Y, Sheng-qing W, Yong W, Reaney MJT, Zhi-min Z, Long-ping Z, Guo S, Yi N, Dong Z, et al. Conversion of poultry manure to biodiesel, a practical method of producing fatty acid methyl esters via housefly (Musca domestica L.) larval lipid. *Fuel* (2017) **210**:463–471. doi:10.1016/j.fuel.2017.08.109
